# A comparison of the utility of the urine dipstick and urine protein-to-creatinine ratio for predicting microalbuminuria in patients with non-diabetic lifestyle-related diseases -a comparison with diabetes

**DOI:** 10.1186/s12882-022-02974-6

**Published:** 2022-11-24

**Authors:** Makoto Ogi, Takuya Seto, Yoshinori Wakabayashi

**Affiliations:** Department of Internal Medicine, Yuurinkouseikai Fuji Hospital, 1784 Niihashi, Gotemba, Shizuoka 412-0043 Japan

**Keywords:** Lifestyle-related disease, Chronic kidney disease (CKD), Urine dipstick, Urine protein-to-creatinine ratio (uPCR), Microalbuminuria

## Abstract

**Background:**

The utility of dipstick proteinuria for predicting microalbuminuria in non-diabetic lifestyle-related diseases compared with the urine protein-to-creatinine ratio (uPCR) and the effect of dipstick proteinuria on the cut-off value (CO) and accuracy of uPCR are unclear.

**Methods:**

The subjects included Japanese patients ≥ 18 years old with lifestyle-related diseases who had an estimated glomerular filtration rate of ≥ 15 ml/min/1.73 m^2^ and uPCR of < 0.5 g/gCr at initiation. Urine dipstick, uPCR and urine albumin-to-creatinine ratio (uACR) were measured three times per case. Microalbuminuria was defined as uACR of 30–299 mg/gCr for at least 2 of 3 measurements. Youden’s Index was used as the optimal CO. Factors associated with microalbuminuria were analyzed using a logistic regression model.

**Results:**

In 313 non-diabetic cases (median 70.8 years old), 3 dipstick proteinuria measurements were independently useful for detecting microalbuminuria, and the CO was set when a trace finding was obtained at least 1 of 3 times (sensitivity 0.56, specificity 0.80, positive predictive value [PPV] 0.73, negative predictive value [NPV] 0.65). A single uPCR measurement was more useful than 3 dipstick measurements, and was useful for detecting microalbuminuria even in cases with three consecutive negative proteinuria findings, indicating that the CO of the second uPCR with G1-3a (*n* = 136) was 0.06 g/gCr (sensitivity 0.76, specificity 0.84. PPV 0.68, NPV 0.89), while that with G3-b4 (*n* = 59) was 0.10 g/gCr (sensitivity 0.56, specificity 0.91. PPV 0.83, NPV 0.71). The sum of 3 uPCRs was useful for detecting microalbuminuria in cases with G1-3a (sensitivity 0.67, specificity 0.94, PPV 0.82, NPV 0.86) and G3b-4 (sensitivity 0.78, specificity 0.94, PPV 0.91 NPV 0.83), with both COs being 0.23 g/gCr. These COs of microalbuminuria did not change when trace or more proteinuria was included, although the sensitivity increased. A high uPCR and low urine specific gravity or creatinine level were independent factors for uACR ≥ 30 mg/gCr in cases with negative proteinuria, although the uPCR was a major predictive factor of a uACR ≥ 30 mg/gCr.

**Conclusions:**

The uPCR (preferably determined using early-morning urine), including in dipstick-negative proteinuria cases with non-diabetic lifestyle-related diseases, can aid in the early detection of microalbuminuria.

**Trial registration:**

Retrospectively registered.

**Supplementary Information:**

The online version contains supplementary material available at 10.1186/s12882-022-02974-6.

## Background

Microalbuminuria is a risk factor of renal dysfunction [1–4], cardiovascular disease [1–6], death [1–3, 5], and dementia [7] in lifestyle-related diseases. Investigation of proteinuria typically begins with the detection of proteinuria via dipstick measurements [8]. The Kidney Disease Improving Global Outcomes (KDIGO) 2012 clinical practice guideline for the evaluation and management of chronic kidney disease recommends the urine albumin-to-creatinine ratio (uACR), urine protein-to-creatinine ratio (uPCR), reagent strip urinalysis for total protein with automated reading, and reagent strip urinalysis for manual reading (in that order) be evaluated for the initial testing of proteinuria [3].

Albuminuria measurement is covered by insurance once every three months in Japan, if incipient diabetic nephropathy is suspected; however, albuminuria measurement is not covered for non-diabetic lifestyle-related diseases. Diabetes and hypertension are the two main causes of end-stage kidney disease worldwide, although the causes in general vary significantly among countries. According to a statistical survey of the Japanese Society for Dialysis Therapy, diabetic nephropathy was the most common primary disease among incident dialysis patients in Japan, followed by nephrosclerosis, the rate of which has been increasing [9].

Lifestyle-related diseases include type 2 diabetes, obesity, metabolic syndrome, non-familial dyslipidemia, hypertension, hyperuricemia, myocardial infarction, stroke, liver disease, cancer, etc., the onset and progression of which are related to lifestyle habits such as eating habits (e.g. excessive intake of salt or sugar), lack of exercise, smoking, and excessive alcohol intake. Lifestyle-related disease had been proposed as a disease name, by clarifying the cause as being an unhealthy lifestyle, and was officially recognized in 1996 in Japan, with this name having been used so that everyone can definitely work on prevention. This study targeted lifestyle-related diseases excluding cancer, etc., that cause CKD such as type 2 diabetes, hypertension, obesity, metabolic syndrome, dyslipidemia, and hyperuricemia.

Advances are also being made with regard to treatment for chronic kidney disease [10]. The prompt detection of microalbuminuria is considered necessary, in order to intervene in patients with lifestyle-related diseases at an early stage.

The urine dipstick is useful for screening uACR ≥ 30 mg/gCr [11–14], with a few limitations having been reported [12, 13]. While it is recommended to diagnose microalbuminuria based on positive results in two of three tests [3], the accuracy and optimal cut-off(CO) for the prediction of microalbuminuria via three dipstick tests are not clear.

Although a CKD classification of A, defined as with a uPCR < 0.15 g/gCr for A1 and 0.15 to 0.49 g/gCr for A2 [2] is more useful for predicting uACR 30-300 mg/gCr than the dipstick test, this is also not sufficient [15]. The uPCR is useful for predicting uACR ≥ 30 mg/gCr [14, 16–18] and microalbuminuria in cases of lifestyle-related diseases [18]. Therefore, in the present study, we compared the utility of dipstick measurements and the uPCR for detecting microalbuminuria. Furthermore, the effect of qualitative proteinuria findings on the optimal CO and accuracy of uPCR in the prediction of microalbuminuria, including its utility for predicting microalbuminuria in proteinuria-negative cases, was unclear, meriting a further examination. In addition to the utility of the uPCR for the detection of microalbuminuria, in cases with negative proteinuria, the effects of urine specific gravity and urine creatinine were also examined.

## Methods

The subjects included Japanese patients ≥ 18 years old who visited Yuurinkousekusei Fuji Hospital from October 2017 to May 2021 for lifestyle-related diseases or CKD due to diseases or aging and who had an estimated glomerular filtration rate (eGFR) of ≥ 15 ml/min/1.73 m^2^ and a uPCR of < 0.5 g/gCr at initiation. Patients with urinary tract infection, nephritis, hereditary renal disease, paraproteinemia, or cancer and kidney transplanted patients were excluded. We interviewed the patients in order to obtain their family history of hereditary diseases, and medical history of infectious diseases and kidney transplants. Urine protein, hematuria, urinary glucose, and urinary sediment were assessed by midstream urine examination for all patients. For those with proteinuria, hematuria, urine dysmorphic erythrocytes or urine cellular casts, ASO, serum IgA, antinuclear antibody, anti-DNA antibody, complements, MPO-ANCA, PR3-ANCA, and cryoglobulin were measured; while those suspected of having nephritis and collagen disease underwent a renal biopsy. Those with proteinuria, hematuria, and purpura, along with IgA vasculitis by skin biopsy were excluded. Serum and urine immunoelectrophoresis was performed in elderly individuals who were positive for proteinuria in order to examine the presence or absence of M protein and Bence-Jones protein, while amyloidosis and multiple myeloma were excluded. An echo examination of the renal urinary tract was performed in all cases and postrenal renal failure, tumors in organs such as kidney, ureter, bladder, and prostate, and polycystic kidney disease were excluded. For cases with negative urine protein and renal dysfunction of unknown causes, tubulointerstitial markers such as urine NAG, urine β2microglobulin, or urine L-FABP were examined, with a renal biopsy performed if necessary, while renal disorders due to interstitial nephritis and sarcoidosis were excluded. A renal biopsy was performed in diabetic patients with proteinuria and hematuria and suspected nephritis, while cases with nephritis were excluded.

A urine dipstick evaluation and uPCR, and uACR measurements were performed in the same urine samples three times per case on different days within one year with the second and third measurements performed using early morning spot urine samples [3]. Patients whose proteinuria had increased to ≥ 0.5 g/gCr at the second or third measurement were not excluded. Patients whose eGFR had decreased by ≥ 30% within 3 months were excluded. The average eGFR during the period was used to determine the GFR category [2].

Negative proteinuria (-) as 0, ( ±) trace as 0.5, ( +) as 1, 2 ( +) as 2, 3 ( +) as 3 were used to determine the dipstick proteinuria score. The sum of the three urine protein dipstick scores for both non-diabetic and diabetic patients showed a significant positive correlation with the sum of the three uPCRs (Supplemental Fig. 1a, b), which was used as the sum of the urine protein dipstick scores (SuPDS).

The three dipstick proteinuria evaluations were classified as proteinuria(-) of 0, proteinuria ≥ ( ±) of 1, or proteinuria( ±)(-) of 0, proteinuria ≥ ( +) of 1, using dummy variables, in order to examine the usefulness of differentiating micro- and macroalbuminuria by qualitative proteinuria using a binomial logistic regression.

The A1 caregory (uPCR < 0.15 g/gCr) is divided into three equal parts: A1L (uPCR 0–0.05 g/gCr), A1M (uPCR 0.06–0.10 g/gCr), and A1H (uPCR 0.11–0.14 g/gCr) in order to examine the association between dipstick proteinuria, uPCR and uACR.

Microalbuminuria was defined as a uACR of 30–299 mg/gCr for at least 2 of 3 measurements, while normoalbuminuria was defined as a uACR of < 30 mg/gCr for at least 2 of 3 measurements, and macroalbuminuria was defined as a uACR of ≥ 300 mg/gCr for at least 2 of 3 measurements. When the uACR was < 30 mg/gCr, 30–299 mg/gCr, and ≥ 300 mg/gCr, once at each measurement, the condition was defined as microalbuminuria in this study.

The c that met the minimum value of [(1 - sensitivity(c))^2^ + (1 - specificity(c))^2^)]^1/2^ of the receiver operating characteristic (ROC) curve was defined as the optimal CO according to the distance (D). The c* that met the maximum of [sensitivity(c*) + specificity(c*) - 1] of ROC curve was defined as the Youden's Index (YI). The optimal CO according to the D and YI were described as CO (D) and CO (YI), respectively [19].

Supplemental Fig. 2a, b shows a graph of the relationship between the CO of uPCR and sensitivity, specificity, YI, and 1—D, which was created from the ROC curve, in order to clarify the effect of the dipstick proteinuria findings on the ability of the uPCR to differentiate uACR ≥ 30 mg/gCr or microalbuminuria.

A urine dipstick was performed to determine the pH, specific gravity, protein, hematuria and glucose levels using a Clinitek Nouvus Automated urine chemistry analyzer (SIEMENS, Berlin, German). Proteinuria was measured using the pyrogallol red method (AR WAKO microTP-AR; FUJIFILM Wako Pure Chemical Industries, Osaka, Japan), urinary albumin was measured using an immunoturbimetric method (Auto Wako Microalbumin; FUJIFILM Wako Pure Chemical Industries), and serum and urine creatinine were measured using the enzyme method (L type WAKO CRE M; FUJIFILM Wako Pure Chemical Corporation) with an autoanalyzer (TBA-FX8, Canon medical systems corporation, Tochigi, Japan).

The correlation between two factors was examined by a simple regression analysis. The relationship between the log uPCR and log median uACR was analyzed by a restricted cubic spline. The chi-squared test, Mann–Whitney U test and Kruskal–Wallis and Steel–Dwass tests were used to compare clinical symptoms and the laboratory values for each category.

The factors differentiating uACR ≥ 30 mg/gCr or microalbuminuria were obtained using the logistic model, while the prediction probability was determined using λ = logit p, *p* = 1/(1 + exp(-λ)).

All statistical analyses were performed using the BellCurve for Excel (Social Survey Research Information Co., Ltd., Tokyo, Japan), SigmaStat Statistics (Systat Software, Inc., CA, USA), and Stata MP version16 (StataCorp LP, College Station, TX, USA) software programs.

## Results

Table 1 shows the patient background. The median age was 70.8 years old for non-diabetic and 68.9 years old for diabetic patients. The BMI was significantly higher in diabetic patients than in non-diabetic patients. Although the BMI and uACR and the BMI and uPCR both exhibited significant positive correlation in non-diabetic patients, no significant correlations were observed in diabetic patients. The abdominal circumference was higher in both men and women in diabetic patients than in non-diabetic patients and a significant positive correlation between abdominal circumference and uPCR & uACR was observed in non-diabetic men, but not in diabetic patients. While uACR and uPCR were higher in hypertensive cases than in non-hypertensive cases among non-diabetic patients, no difference was observed in diabetic patients. Classified by G stage, both uACR and uPCR increased as the stage progressed in diabetic patients, with uACR and uPCR significantly higher in G3a and G3b in diabetic patients than in non-diabetic patients. The uACR and uPCR increased significantly with the increase in urinary protein qualitative findings, in both diabetic and non-diabetic patients.

**Table 1:** Characteristics of patients

			Non-diabetes						Diabetes				*P* Value§		
continuous variables	n		regression equation		R	*P* value	n		regression equation		R	*P* value			
Age (years)	313	70.8 (60.0–79.4)	uACR = -0.5779 × age + 96.32		0.126	0.026	131	68.9 (60.5–76.9)	uACR = 0.7831 × age + 6.53		0.142	0.106	0.454		
			uPCR = -0.00067 × age + 0.1464		0.106	0.062			uPCR = 0.00230 × age-0.04744		0.269	0.0019			
BMI	313	24.1(21.9–26.7)	uACR = 3.4360 × BMI-27.51		0.227	5.0E-05	131	24.8(22.8–27.6)	uACR = -1.5507 × BMI + 100.07		0.113	0.197	0.008		
			uPCR = 0.003843 × BMI + 0.0062		0.183	0.0011			uPCR = -0.00364 × BMI + 0.2034		0.171	0.050			
Waist circumference(WC)(cm)															
male	167	88.0(82.5–94.3)	uACR = 1.4548 × WC-69.71		0.205	0.0078	77	90.5(85.0–99.5)	uACR = -0.4629 × WC + 100.50		0.084	0.467	0.042		
			uPCR = 0.001589 × WC-0.0373		0.167	0.030			uPCR = -0.00086 × WC + 0.1903		0.102	0.377			
female	121	83.0(76.0–89.5)	uACR = 0.1405 × WC + 38.46		0.035	0.69	42	90.0(86.3–96.0)	uACR = -0.8046 × WC + 135.52		0.155	0.327	0.0001		
			uPCR = 8.96E-5 × WC + 0.0834		0.015	0.87			uPCR = -0.00213 × WC + 0.2980		0.279	0.074			
Time between measurements(day)															
1–2	313	70.0 (42.0–98.0)					131	63.0 (42.0–84.0)					0.034		
2–3	313	70.0 (49.0–91.0)					131	63.0 (54.5–81.5)					0.034		
			Non-diabetes						Diabetes				PII	P¶	P¶
categorical variables		n	uACR(mg/gCr)	uPCR(g/gCr)				n	uACR(mg/gCr)	uPCR(g/gCr)			n	uACR	uPCR
Age															
< 70 years old		150	27.5(14–73.8)	0.07(0.03–0.14)				70	36(13–62.5)	0.06(0.03–0.12)**			0.34	0.91	0.47
≥ 70 years old		163	36(16–75.5)	0.08(0.04–0.14)				61	43(23–87)	0.11(0.05–0.19)				0.18	0.034
Sex															
male		184	31(13.3–76.8)	0.07(0.04–0.14)				84	33(13–72.3)	0.085(0.04–0.15)			0.051	0.84	0.53
female		129	36(16.5–73.5)	0.08(0.035–0.14)				47	42(23–89)	0.07(0.03–0.16)				0.22	0.72
BMI															
< 25		189	25(13–69)**	0.06(0.03–0.14)*				66	40.5(14.5–94)	0.10(0.043–0.16)			0.066	0.052	0.038
≥ 25		124	38(18–96)	0.08(0.05–0.15)				65	36(17–64)	0.06(0.03–0.13)				0.27	0.13
Current smoking															
( +)		41(13.1)	28(15–122)	0.09(0.04–0.16)				17(13.0)	35(13–61)	0.07(0.03–0.12)			0.905	0.52	0.40
(-)			34(15–73.3)	0.07(0.038–0.14)					38.5(17.3–78)	0.08(0.04–0.16)				0.23	0.26
Complicated disease		n(%)													
Hypertension															
( +)		216(69.0)	38.5(17–84.5)***	0.09(0.04–0.15)**				94(71.8)	36(18–77.5)	0.08(0.04–0.158)			0.565	0.76	0.81
(-)			20(11–41)	0.06(0.03–0.10)					37(12–87)	0.07(0.03–0.13)				0.072	0.14
Dyslipidemia															
( +)		181(57.8)	36(15–75)	0.07(0.04–0.14)				76(58.0)	34(14.8–72.3)	0.08(0.04–0.135)			0.971	0.88	0.76
(-)			28(14.7–75.3)	0.07(0.03–0.15)					41(20–101.5)	0.08(0.035–0.15)				0.11	0.52
Hyperuricemia															
( +)		122(39.0)	33(11.3–85)	0.075(0.033–0.148)				35(26.7)	45(21–98)	0.12(0.05–0.18)			0.014	0.093	0.22
(-)			34(16–70)	0.07(0.04–0.14)					34.5(13–72.3)	0.07(0.03–0.135)				0.99	0.9
GFR category^c^			*P* = 0.049^a^	*P* = 0.18					*P* = 1.4E-0.9	*P* = 2.5E-12					
1		6(1.9)	68(45.3–98.3)	0.095(0.058–0.148)				12(9.2)	26(11.5–58.8)	0.07(0.04–0.128)			< 0.0001	0.044	0.63
2		89(28.4)	34(15–77)	0.07(0.04–0.14)				57(43.5)	26(11.5–60)	0.05(0.03–0.115)				0.058	0.013
3a		118(37.7)	24.5(12.3–58)	0.06(0.03–0.128)				27(20.6)	38(23–76)^b2^	0.08(0.05–0.15)^b2^				0.00094	0.014
3b		66(21.1)	47(14.3–102.5)	0.085(0.043–0.148)				24(18.3)	68(36.8–138.8)^a2b2c1^	0.16(0.095–0.265)^a2b2c2^				0.0081	2.5E-05
4		34(10.9)	40(18.3–91.5)	0.11(0.06–0.158)				11(8.4)	48(24–108)^a1b1^	0.08(0.05–0.19)^b1d1^				0.42	0.89
1st dipstick proteinuria			*P* = 5.6E-12^a^	*P* = 4.4E-19					*P* = 3.5E-06	*P* = 3.0E-06					
(-)		233(74.4)	24(13–57)	0.06(0.03–0.10)				99(75.6)	28(13–57.5)	0.06(0.03–0.12)			0.967	0.48	0.35
( ±)		46(14.7)	63.5(25.3–106.5)	0.145(0.093–0.20)				17(13.0)	86(41–134)	0.13(0.11–0.21)				0.39	0.89
( +)		30(9.6)	105(63.5–172)	0.17(0.103–0.24)				13(9.9)	108(83–197)	0.21(0.13–0.26)				0.52	0.54
2( +)		4(1.3)	252(230.3–260.8)	0.365(0.318–0.403)				2(1.5)	223(131.3–315.8)	0.32(0.18–0.46)				1.00	1.00
3( +)		0(0)						0(0)							
1st uPCR (g/gCr)															
0–0.14		238(76.0)	*P* = 3.3E-33^a^	*P* = 1.1E-61				95(72.5)	*P* = 3.5E-17	*P* = 1.1E-25			0.032		
≦0.05		95(30.4)	15(8–25.3)	0.03(0.01–0.04)				49(37.4)	13(10–24)	0.03(0.02–0.04)				1.00	0.49
0.06—0.10		106(33.9)	30(17–53)	0.07(0.06–0.09)				26(19.8)	32(23.8–41.5)	0.07(0.06–0.088)				0.74	0.54
0.11—0.14		37(11.8)	67(33–80)	0.13(0.12–0.14)				20(15.3)	50(40.3–61.8)	0.12(0.11–0.13)				0.32	0.011
0.15–0.49		75(24.0)	122(81.5–177.5)	0.20(0.16–0.26)				36(27.5)	112.5(77.5–177)	0.21(0.17–0.26)				0.90	0.46
1st uACR (mg/gCr)			*P* = 5.8E-52^a^	*P* = 8.5E-25					*P* = 1.4E-21^b^	*P* = 1.6E-13					
< 30		149(47.6)	14(9–19)	0.04(0.02–0.07)				55(42.0)	13(10–21.5)	0.04(0.02–0.06)			0.530	0.59	0.41
30–299		161(51.4)	73(47–115)	0.13(0.08–0.19)				75(57.3)	68(43–110.5)	0.13(0.09–0.21)				0.65	0.37
≧300		3(1.0)	323(323–392.5)	0.45(0.42–0.47)				1(0.8)							
			*P* = 1.3E-39^a^	*P* = 9.2E-24					*P* = 2.2E-14^b^	*P* = 3.3E-10					
normoalbuminuria		158(50.5)	15(9–23.8)	0.045(0.02–0.078)				54(41.2)	13.5(10–26.8)	0.04(0.03–0.068)			0.040	0.70	0.69
microalbuminuria		149(47.6)	71(40–117)	0.13(0.07–0.19)				77(58.8)	63(36–112)	0.13(0.07–0.21)				0.41	0.83
macroalbuminuria		6(1.9)	241.5(162.3–314)	0.36(0.25–0.44)				0(0)							

Microalbuminuria: uACR(urine albumin-to-creatinine ratio) 30–299 mg/gCr at least twice for 3 measurements

Data are presented as the median (interquartile range)

*GFR* Glomerular filtration rate, *BMI* Body mass index, *uPCR* Urine protein-to-creatinine ratio, *uACR* Urine albumin-to-creatinine ratio

^a^Kruskal-Wallis test was used to compare items in the same column

^b^The Mann–Whitney's U test was used to compare items in the same column

^c^Comparison of uACR and uPCR of GFR category: ^a1^*p* < 0.05 vs G1, ^a2^*p* < 0.01 vs G1, ^b1^*p* < 0.05 vs G2, ^b2^*p* < 0.05 vs G2, ^c1^*p* < 0.05 vs G3a, ^c2^*p* < 0.01 vs G3a, ^d1^*p* < 0.05 vs G3b by the Steel–Dwass test

^§^The Mann–Whitney's U test was used to compare continuous variables of non-diabetes and diabetes

IIThe chi-square test was used to compare items of non-diabetes and diabetes

^¶^The Mann–Whitney's U-test was used to compare items of non-diabetes and diabetes

^*^P < 0.05,**p < 0.01, ***p < 0.001 by the Mann–Whitney's U-test test comparing the items in the same column

Supplemental Figs. 3a, b show the distribution of dipstick findings by the uPCR and uACR in non-diabetic and diabetic patients. Supplemental Table 1 shows that the uPCR and uACR increased as the qualitative urinary findings increased in non-diabetic patients. The uACR became higher as it increased to A1L, A1M, A1H, and A2 within the same qualitative proteinuria level. Although uACR increased as the qualitative proteinuria increased in A2, there was no difference in A1L, A1M, and A1H in non-diabetic patients. As qualitative proteinuria increased in G1-3a and G3b-4, the uACR ≥ 30 mg/gCr increased in the uPCR high fraction (*p* = 9.73E-11, 2.79E-9), whereas the uACR < 30 mg/gCr or less, in contrast, increased in the uPCR low fraction of qualitative proteinuria (-) (*P* = 1.33E-8, 0.002) in non-diabetic patients. The distribution of the number of uACR < or ≥ 30 mg/gCr by uPCR category was tested by χ2 between G1-3a and G3b4, indicating a significant difference in the number of uACR < 30 mg/gCr, ≥ 30 mg/gCr of proteinuria (-), and the number of uACR ≥ 30 mg/gCr of proteinuria ( ±) in non-diabetic patients (*P* = 0.043, 0.033, 0.022).

### A comparison of the utility of the urine dipstick and uPCR for predicting uACR ≥ 30 mg/gCr

Supplemental Table 2 indicates that a single dipstick for non-diabetic and diabetic patients was useful for predicting uACR ≥ 30 mg/gCr, and the CO (D, YI) was trace proteinuria.

Supplemental Fig. 4a, b indicate that in non-diabetic and diabetic patients, the uPCR was more useful for differentiating uACR ≥ 30 mg/gCr than a dipstick evaluation (*p* < 0.0001, respectively).

Table 2 indicates that the uPCR is useful for differentiating between uACR < 30 mg/gCr and ≥ 30 mg/gCr at all qualitative proteinuria levels and the area under the receiver operating characteristic curve (AUC) increased with higher qualitative proteinuria findings in both non-diabetic and diabetic patients.

**Table 2:** Discrimination of uACR ≥ 30 mg/gCr and < 30 mg/gCr by uPCR according to the dipstick proteinuria in non-diabetic and diabetic patients

**Cause**							**CO**^**a**^ of						
of CKD	G stage	dipstick urine protein	number	AUC	SE	*P* Value	uPCR (g/gCr)	Sensitivity	Specificity	PPV	NPV	Distance	Se + Sp-1
Non-diabetes	G1-4	(-)	724	0.817	0.017	< 0.001	0.07	0.709	0.826	0.741	0.801	0.339	0.535
		(-)( ±)	843	0.839	0.014	< 0.001	0.07	0.772	0.797	0.766	0.802	0.305	0.569
		(-)( ±)( +)	918	0.857	0.013	< 0.001	0.07	0.806	0.789	0.792	0.803	0.287	0.595
							0.09	0.706	0.891	0.866	0.752	0.314	0.597
		(-)( ±)( +)2( +)3( +)	939	0.863	0.012	< 0.001	0.07	0.815	0.789	0.801	0.803	0.281	0.603
							0.09	0.719	0.891	0.873	0.752	0.302	0.610
Non-diabetes	G1-3a	(-)	507	0.795	0.022	< 0.001	0.06	0.741	0.755	0.642	0.830	0.357	0.495
							0.07	0.677	0.843	0.719	0.815	0.359	0.520
		(-)( ±)	578	0.820	0.019	< 0.001	0.07	0.741	0.821	0.750	0.814	0.315	0.562
		(-)( ±)( +)	629	0.843	0.017	< 0.001	0.07	0.784	0.817	0.786	0.814	0.284	0.600
		(-)( ±)( +)2( +)	639	0.849	0.016	< 0.001	0.07	0.791	0.817	0.793	0.814	0.278	0.607
Non-diabetes	G3b-4	(-)	217	0.847	0.026	< 0.001	0.07	0.764	0.776	0.778	0.761	0.326	0.539
							0.10	0.636	0.907	0.875	0.708	0.375	0.543
		(-)( ±)	265	0.864	0.022	< 0.001	0.09	0.735	0.864	0.871	0.723	0.298	0.599
							0.10	0.721	0.881	0.883	0.717	0.303	0.602
		(-)( ±)( +)	289	0.875	0.020	< 0.001	0.10	0.756	0.876	0.894	0.721	0.274	0.632
		(-)( ±)( +)2( +)3( +)	300	0.883	0.019	< 0.001	0.10	0.771	0.876	0.902	0.721	0.260	0.647
Diabetes	G1-4	(-)	308	0.860	0.022	< 0.001	0.07	0.745	0.865	0.844	0.775	0.289	0.610
		(-)( ±)	355	0.873	0.019	< 0.001	0.07	0.794	0.839	0.856	0.771	0.262	0.632
		(-)( ±)( +)	389	0.889	0.017	< 0.001	0.07	0.824	0.833	0.874	0.771	0.243	0.657
		( +)( ±)( +)2( +)	393	0.891	0.017	< 0.001	0.07	0.827	0.833	0.876	0.771	0.240	0.660
Diabetes	G1-3a	(-)	245	0.835	0.028	< 0.001	0.07	0.721	0.866	0.816	0.789	0.310	0.586
		(-)( ±)	274	0.855	0.024	< 0.001	0.07	0.772	0.848	0.833	0.791	0.274	0.620
		(-)( ±)( +)	288	0.865	0.023	< 0.001	0.07	0.792	0.842	0.843	0.791	0.261	0.634
Diabetes	G3b-4	(-)	63	0.931	0.031	< 0.001	0.07	0.810	0.857	0.919	0.692	0.238	0.667
							0.06	0.929	0.762	0.886	0.842	0.249	0.690
		(-)( ±)	81	0.906	0.035	< 0.001	0.07	0.845	0.783	0.907	0.667	0.267	0.627
		(-)( ±)( +)	101	0.928	0.027	< 0.001	0.07	0.885	0.783	0.932	0.667	0.246	0.667
		(-)( ±)( +)2( +)	105	0.932	0.026	< 0.001	0.07	0.890	0.783	0.936	0.667	0.244	0.673

*CKD* Chronic kidney disease, *uPCR* urine protein-to-creatinine ratio, *uACR* urine albumin-to-creatinine ratio

*AUC* Area under curve, *SE* Standard error, *CO* Cutoff value, *Se* Sensitivity, *Sp* Specificity, *PPV* Positive predictive value, *NPV* Negative predictive value

^a^CO of minimum distance on the ROC curve closest to the (0,1) or Youden's Index

Supplemental Fig. 5a shows that the CO (YI) of uPCR for detecting uACR ≥ 30 mg/gCr was 0.07 g/gCr with dipstick proteinuria(-) and (-)( ±) and 0.09 g/gCr with dipstick proteinuria(-)( ±)( +) and (-)( ±)( +)2,3( +) in non-diabetic patients of G1-4. Supplemental Fig. 5b shows that the ratio of sensitivity in patients with qualitative proteinuria (-)( ±) or more versus (-) increased with the increase in the uPCR, and the higher the qualitative proteinuria findings, the higher the ratio. In contrast, the ratio of specificity hardly changed with the increase in the uPCR.

**Fig. 1:**
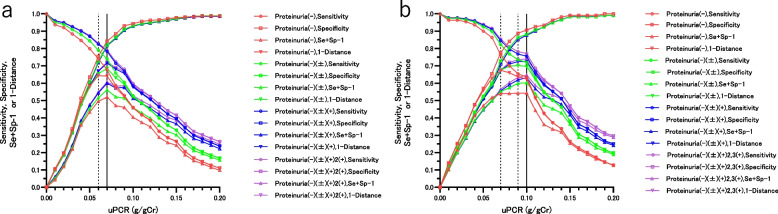
**a** The utility of the cut-off values of the uPCR and sensitivity, specificity, Se + Sp-1, and 1—Distance in the differentiation of uACR ≥ 30 mg/gCr in non-diabetic patients with G stages 1 to 3a according to dipstick proteinuria. Red: patients with proteinuria(-), Green: patients with proteinuria(-)( ±), Blue: patients with proteinuria(-)( ±)( +), Purple patients with proteinuria(-)( ±)( +)2( +),○: Sensitivity, □: Specificity, △: Se + Sp-1, ▽: 1—Distance. The solid vertical line indicates a CO (YI) of 0.07 g/gCr at dipstick proteinuria(-). The CO (YI) does not change even if more than a trace of proteinuria is added. The dotted vertical line indicates a CO (D) of 0.06 g/gCr at dipstick proteinuria(-). The CO (D) matches the CO (YI) when trace or more proteinuria is included. **b** The utility of the cut-off values of the uPCR and sensitivity, specificity, Se + Sp-1, and 1- Distance in the differentiation of uACR ≥ 30 mg/gCr in non-diabetic patients with G stages 3b to 4 according to dipstick proteinuria. Red: patients with proteinuria(-), Green: patients with proteinuria(-)( ±), Blue: patients with proteinuria(-)( ±)( +), Purple patients with proteinuria(-)( ±)( +)2,3( +),○: Sensitivity, □: Specificity, △: Se + Sp-1, ▽: 1—Distance. The solid vertical line indicates a CO (YI) of 0.10 g/gCr at dipstick proteinuria(-). The CO (YI) does not change even if dipstick proteinuria ( ±) or more is included. The dotted vertical line indicates a CO(D) of 0.07 g/gCr at dipstick proteinuria(-) and 0.09 g/gCr at proteinuria(-)( ±). The CO (D) matches the CO (YI) when dipstick proteinuria is (-) ( ±) ( +) or more. The results are shown in Table 2.

Figure 1a, b show that, in non-diabetic patients with G1-3a and G3b4 and proteinuria (-), the CO (YI) for detecting uACR ≥ 30 mg/gCr was 0.07 g/gCr and 0.10 g/gCr, respectively, and did not change even if qualitative proteinuria ( ±) or worse was included. The CO (D) of non-diabetic patients with G1-3a and G3b-4 and proteinuria (-) were 0.06 g/gCr and 0.07 g/gCr, respectively, and when qualitative proteinuria ( ±), or ( ±) and higher was included, it matched the CO (YI).

Supplemental Fig. 6a, b shows that, when G1-3a and 3b-4 were compared in non-diabetic patients, the CO (YI) for detecting uACR ≥ 30 mg/gCr in G1-3a was 0.07 g/gCr, with a higher specificity than that for G3b-4, while the CO (YI) in G3b-4 was 0.10 g/gCr, with a higher sensitivity than that for G1-3a.

The CO (D or YI) for detecting uACR ≥ 30 mg/gCr was 0.07 g/gCr for all qualitative proteinuria levels in diabetic patients with G1-4, G1-3a and G3b-4 (Table 2).

### A comparison of the utility of the urine dipstick and uPCR for predicting microalbuminuria

**Fig. 2:**
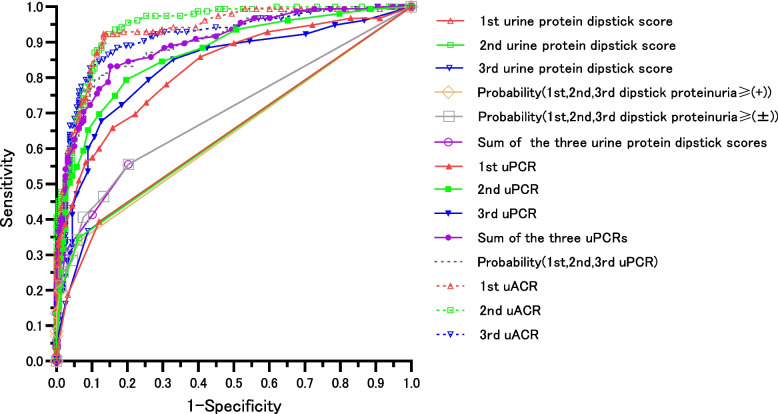
The ROC curve for the differentiation of normoalbuminuria and micro- and macroalbuminuria using the urine protein dipstick score, urine protein-to-creatinine ratio, and urine albumin-to-creatinine ratio in non-diabetic patients. Probability (1st, 2nd, 3rd dipstick proteinuria ≥ ( +)): Probability of micro- and macroalbuminuria based on the logistic model using dummy variable dipstick proteinuria ≥ ( +): 1, dipstick proteinuria( ±) (-): 0. Probability (1st, 2nd, 3rd dipstick proteinuria ≥ ( ±)): Probability of micro- and macroalbuminuria based on the logistic model using dummy variable dipstick proteinuria ≥ ( ±):1, dipstick proteinuria(-):0. Probability (1st, 2nd, 3rd uPCR): Probability of micro- and macroalbuminuria based on the logistic model using 1st, 2nd, and 3rd uPCR. The detailed results are listed in Table 3

Figure 2, Table 3 and Supplemental Table 3 indicate that, for both non-diabetic and diabetic patients with G1 to 4 and all qualitative proteinuria findings, three dipstick proteinuria evaluations were useful for differentiating microalbuminuria, respectively, and the CO was trace proteinuria.

**Table 3:** A comparison of the ability to predict microalbuminuria or worse using the dipstick proteinuria, uPCR, or uACR in non-diabetic patients with G1-4 and all qualitative proteinuria findings

	AUC	SE	*P* Value	CO^f^	Sensitivity	Specificity	PPV	NPV	Distance	Se + Sp-1	*P* Value vs	*P* Value vs Sum of	*P* Value vs Sum of
											P[1,2,3dipstick ≥ ( ±)]^b^	1,2,3dipstick scores^c^	1,2,3uPCR
1st urine protein dipstick score	0.642	0.024	< 0.001	0.5^ g^	0.394	0.880	0.763	0.597	0.618	0.273	0.0015	0.0003	< 0.0001
2nd urine protein dipstick score	0.647	0.021	< 0.001	0.5	0.348	0.937	0.844	0.594	0.655	0.285	0.0023	0.0039	< 0.0001
3rd urine protein dipstick score	0.643	0.022	< 0.001	0.5	0.368	0.911	0.803	0.595	0.638	0.279	0.0013	0.0011	< 0.0001
Probability[1,2,3dipstick ≥ ( +)]^a^	0.643	0.022	< 0.001	0.650	0.342	0.930	0.828	0.590	0.662	0.272	0.0032	0.0008	< 0.0001
Probability[1,2,3dipstick ≥ ( ±)]^b^	0.701	0.026	< 0.001	0.532	0.555	0.797	0.729	0.646	0.489	0.352	-	0.801	< 0.0001
Sum of 1,2,3dipstick scores^c^	0.702	0.025	< 0.001	0.5	0.555	0.797	0.729	0.646	0.489	0.352	0.801	-	< 0.0001
1st uPCR (g/gCr)	0.826	0.023	< 0.001	0.08	0.729	0.747	0.739	0.738	0.371	0.476	< 0.0001	< 0.0001	< 0.0001
				0.10	0.658	0.842	0.803	0.715	0.377	0.500			
2nd uPCR (g/gCr)	0.867	0.020	< 0.001	0.07	0.794	0.804	0.799	0.799	0.285	0.597	< 0.0001	< 0.0001	0.034
3rd uPCR (g/gCr)	0.831	0.024	< 0.001	0.07	0.794	0.741	0.750	0.785	0.332	0.534	< 0.0001	< 0.0001	0.00018
				0.09	0.677	0.873	0.840	0.734	0.347	0.551			
Sum of 1,2,3uPCR (g/gCr)^d^	0.898	0.017	< 0.001	0.23	0.832	0.848	0.843	0.838	0.226	0.680	< 0.0001	< 0.0001	-
Probability(1,2,3uPCR)^e^	0.900	0.017	< 0.001	0.438	0.806	0.880	0.868	0.822	0.228	0.686	< 0.0001	< 0.0001	0.470
1st uACR (mg/gCr)	0.932	0.014	< 0.001	30	0.923	0.867	0.872	0.919	0.154	0.790	< 0.0001	< 0.0001	0.091
2nd uACR (mg/gCr)	0.946	0.012	< 0.001	30	0.923	0.854	0.861	0.918	0.165	0.777	< 0.0001	< 0.0001	0.0094
3rd uACR (mg/gCr)	0.915	0.017	< 0.001	30	0.845	0.880	0.873	0.853	0.196	0.725	< 0.0001	< 0.0001	0.410
				31	0.826	0.905	0.895	0.841	0.198	0.731			

*uPCR* urine protein-to-creatinine ratio, *uACR* urine albumin-to-creatinine ratio, *PPV* Positive predictive value, *NPV* Negative predictive value, *Se* Sensitivity, *Sp* Specificity

^a^Probability of micro- and macroalbuminuria by a logistic model using the 1st, 2nd and 3rd dipstick urine protein. Dammy variable dipstick proteinuria ≥ ( +);1 and ( ±)(-);0, respectively. Supplemental Table 4

^b^Probability of micro- and macroalbuminuria by a logistic model using the 1st, 2nd and 3rd dipstick urine protein. Dammy variable dipstick proteinuria ≥ ( ±);1 and (-);0, respectively. Supplemental Table 4, 5

^c^Sum of the 1st, 2nd and 3rd urine protein dipstick scores. Urine protein dipstick score were defined as dipstick(-):0,( ±):0.5, ( +):1, 2( +):2, and 3( +):3, respectively

^d^Sum of the 1st, 2nd,and 3rd uPCR

^e^Probability of micro- and macroalbuminuria by a logistic model using the 1st, 2nd, and 3rd uPCRs. Supplemental Table 6

^f^CO of minimum distance on the ROC curve closest to the (0,1) or Youden's Index

^g^CO 0.5 on the dipstick test denotes dipstick proteinuria trace

When proteinuria 1( +) or higher was used as the CO, there was no marked difference in the AUC between one dipstick evaluation and the probability using three dipstick measurements in non-diabetic patients. Supplemental Table 4 indicate that three dipstick proteinuria measurements were independently useful for detecting microalbuminuria in the logistic model when trace proteinuria was used as the CO in non-diabetic patients.　When trace proteinuria was used as the CO, the probability of microalbuminuria according to a logistic model using the three dipstick proteinuria measurements was more useful for detecting microalbuminuria than a single dipstick evaluation in non-diabetic patients (Table 3, Supplemental Table 5). Associations between logit p and the probability of microalbuminuria were shown in Supplemental Fig. 7. The SuPDS was equal to the probability of microalbuminuria using proteinuria according to the three dipstick measurements with trace proteinuria as the CO for detecting microalbuminuria, with the CO set at a trace finding being obtained at least once and it was also found more useful than a single dipstick test in non-diabetic patients (Table 3).

Each single uPCR was useful for differentiating microalbuminuria, with one uPCR measurement being more useful for differentiating microalbuminuria than the probability of microalbuminuria using proteinuria according to the three dipstick measurements with trace proteinuria as the CO for detecting microalbuminuria or SuPDS in both non-diabetic and diabetic patients (Table 3, Supplemental Table 3).

Supplemental Table 6 shows that three uPCR measurements were independently useful for detecting microalbuminuria via a logistic model in both non-diabetic and diabetic patients. The predictive probability based on the three uPCR measurements was as useful for differentiating microalbuminuria as the sum of three uPCRs and more useful than one uPCR measurement (Table 3, Supplemental Table 3).

Supplemental Table 7 indicates that, for SuPDS0 in non-diabetic and diabetic patients, a second uPCR measurement was useful for differentiating microalbuminuria, and the CO (YI) of the second uPCR was 0.06 g/gCr in G1-3a and 0.10 g /gCr in G3b-4 in non-diabetic patients. At SuPDS (0,0.5) or more, the CO (YI) of the second uPCR in G1-3a was 0.07 g/gCr, and that in G3b-4 did not change in non-diabetic patients. At SuPDS0 and (0, 0.5), the CO (D, YI) of the second uPCR was 0.06 g/gCr in diabetic patients with G1-4.

**Fig. 3:**
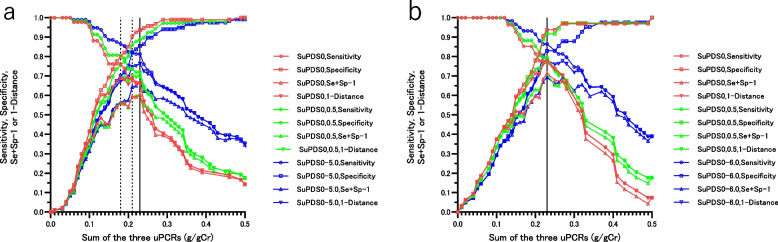
**a** The utility of the cut-off values of the sum of the three uPCRs and sensitivity, specificity, Se + Sp-1, and 1—Distance in the differentiation of normoalbuminuria and micro- and macroalbuminuria in non-diabetic patients with G stages 1 to 3a according to the three SuPDS (sum of the three urine protein dipstick scores). Red: SuPDS0, all three urine protein dipsticks were negative; Green: SuPDS0 or 0.5, all three urine protein dipsticks were negative, or two urine protein dipstick were negative and one urine protein dipstick was trace; Blue: SuPDS0-5.0. ○: Sensitivity, □: Specificity, △: Se + Sp-1, ▽: 1—Distance. The solid vertical line indicates that the CO (YI) of the sum of the three uPCRs with SuPDS0, 0 or 0.5, or 0–5.0 was 0.23 g/gCr. The dotted vertical lines indicate that the CO (D) of the sum of the three uPCRs with SuPDS0 was 0.18 g/gCr, that with SuPDS0 or 0.5 was 0.21 g/gCr. The CO (D) of the sum of the three uPCRs with SuPDS0-5.0 coincided with the CO (YI) at 0.23 g/gCr. **b** The utility of the cut-off values of the sum of the three uPCRs and the sensitivity, specificity, Se + Sp-1, and 1—Distance in the differentiation of normoalbuminuria and micro- and macroalbuminuria in non-diabetic patients with G stage 3b to 4 according to the three SuPDS. Red: SuPDS0; Green: SuPDS0 or 0.5; Blue: SuPDS0-6.0. ○: Sensitivity, □: Specificity, △: Se + Sp-1, ▽: 1—Distance. The solid vertical line indicates that the CO (D and YI) of the sum of the three uPCRs with SuPDS0, 0 or 0.5, or 0–6.0 was 0.23 g/gCr. The results are shown in Table 4

Figure 3a, b and Table 4 show that, for SuPDS0, the sum of the three uPCRs was useful for differentiating microalbuminuria, with a CO (YI) of 0.23 g/gCr for both G1-3a and G3b-4, and the CO (YI) did not change, even at SuPDS (0,0.5) or more in non-diabetic patients. The CO (D) of non-diabetic patients with G1-3a and SuPDS0 was 0.18 g/gCr, which matched the CO (YI) at SuPDS (0–5.0) in non-diabetic patients. In diabetic patients, the CO (YI) of SuPDS 0, 0–0.5 was 0.23 g/gCr, but at SuPDS 0–4.0, the CO (YI) increased to 0.28 g/gCr.

**Table 4:** Prediction of microalbuminuria or worse based on the sum of three uPCR values according to the G stage and the sum of three urine protein dipstick scores in non-diabetic and diabetic patients

		Sum of the three urine protein dipstick scores^a^					CO of sum of the three uPCRs (g/gCr)^d^							*P* Value^e^ vs			*P* Value^f^ vs		
	G stage		n	AUC	SE	*P*-Value		Sensitivity	Specificity	PPV	NPV	Distance	Se + Sp-1	1st uPCR	2 nd uPCR	3rd uPCR	1st uACR	2nd uACR	3rd uACR
Non-diabetes	G1-4	0^b^	195	0.876	0.027	< 0.001	0.23	0.710	0.937	0.860	0.855	0.297	0.647	< 0.0001	0.068	0.004	0.48	0.029	0.91
		0,0.5^c^	233	0.876	0.024	< 0.001	0.23	0.747	0.894	0.819	0.847	0.274	0.642	< 0.0001	0.097	0.003	0.29	0.0074	0.63
		0–6.0	313	0.898	0.017	< 0.001	0.23	0.832	0.848	0.843	0.838	0.226	0.680	< 0.0001	0.034	0.0002	0.091	0.0094	0.41
Non-diabetes	G1-3a	0	136	0.855	0.037	< 0.001	0.18	0.762	0.798	0.627	0.882	0.312	0.560	< 0.0001	0.77	0.005	0.15	0.0082	0.16
							0.23	0.667	0.936	0.824	0.863	0.339	0.603						
		0,0.5	163	0.857	0.032	< 0.001	0.21	0.737	0.858	0.737	0.858	0.299	0.595	< 0.0001	0.69	0.005	0.12	0.0025	0.99
							0.23	0.719	0.887	0.774	0.855	0.303	0.606						
		0–5.0	213	0.885	0.023	< 0.001	0.23	0.813	0.855	0.821	0.847	0.237	0.667	< 0.0001	0.95	0.0004	0.076	0.0037	0.99
Non-diabetes	G3b-4	0	59	0.895	0.043	< 0.001	0.23	0.778	0.938	0.913	0.833	0.231	0.715	0.072	0.006	0.42	0.79	0.87	0.27
		0,0.5	70	0.905	0.036	< 0.001	0.23	0.794	0.917	0.900	0.825	0.222	0.711	0.047	0.005	0.24	0.92	0.73	0.18
		0–6.0	100	0.915	0.027	< 0.001	0.23	0.864	0.829	0.879	0.810	0.218	0.694	0.063	0.001	0.25	0.48	0.85	0.031
Diabetes	G1-4	0	85	0.834	0.048	< 0.001	0.20	0.806	0.796	0.744	0.848	0.282	0.601	0.021	0.011	0.48	0.97	0.062	0.16
							0.23	0.750	0.857	0.794	0.824	0.288	0.607						
		0,0.5	102	0.863	0.038	< 0.001	0.23	0.816	0.811	0.800	0.827	0.263	0.628	0.012	0.004	0.27	0.91	0.11	0.14
		0–4.0	131	0.903	0.027	< 0.001	0.26	0.792	0.889	0.910	0.750	0.236	0.681	0.003	0.001	0.32	0.75	0.17	0.17
							0.28	0.779	0.907	0.923	0.742	0.239	0.687						

*uPCR* urinary protein-to-creatinine ratio, u*ACR* urinary albumin-to-creatinine ratio, *AUC* Area under the curve, *SE* Standard error, *CO* Cut-off value

*PPV* Positive predictive value, *NPV* Negative predictive value, *Se* Sensitivity, *Sp* Specificity

1.2.3 uPCR denotes the sum of the three uPCRs

^a^urine protein dipstick score: (-) = 0,( ±) = 0.5, ( +) = 1, 2( +) = 2, 3( +) = 3

^b^Dipstick proteinuria is negative three times in a row

^c^Dipstick proteinuria is negative three times in a row: or is negative two out of three times, with once being traced

^d^CO of the minimum distance on the ROC curve closest to the (0,1) or Youden's Index

^e^Comparison of the utility of a single uPCR and the sum of three uPCR for predicting microalbuminuria

^f^Comparison of the utility of the sum of three uPCR and a single ACR for predicting microalbuminuria

Microalbuminuria: uACR(urine albumin-to-creatinine ratio) 30–299 mg/gCr at least twice for 3 measurements

**Factors predicting uACR ≥ 30 mg/gCr in patients with dipstick-negative proteinuria.**

Supplemental Table 8 show that, on comparing the background factors for uACR ≥ 30 mg/gCr and < 30 mg/gCr in non-diabetic patients with proteinuria (-), those with uACR ≥ 30 mg/gCr had an older age, less frequent male sex, more hypertension, more advanced G stage, higher urinary pH, and significantly lower urine specific gravity (uSG) and urine creatinine (uCr) than those with uACR < 30 mg/gCr. The background factors for diabetes and proteinuria (-) were similar.

Supplemental Table 9 shows that, among factors with *p* < 0.01, the uPCR and uCr and uSG were extracted from an examination of factors predicting uACR ≥ 30 mg/gCr via a logistic model. Supplemental Table 10 shows that the uPCR and uCr and the uPCR and uSG were independently useful for predicting uACR ≥ 30 mg/g in non-diabetic and diabetic patients.

**Fig. 4:**
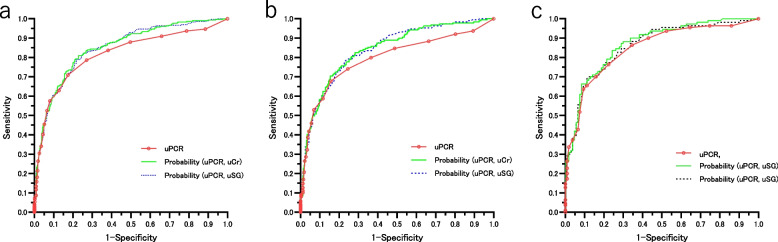
**a** Differentiation of uACR ≥ 30 mg/gCr and < 30 mg/gCr by the uPCR, Probability (uPCR, uCr), and Probability (uPCR, uSG) in non-diabetic patients with G stages 1 to 4 and dipstick-negative proteinuria by the ROC curve. Probability (uPCR, uCr): Probability of uACR ≥ 30 mg/gCr based on the logistic model using the uPCR and uCr, Probability (uPCR, uSG): Probability of uACR ≥ 30 mg/gCr based on the logistic model using the uPCR and uSG. **b** Differentiation of uACR ≥ 30 mg/gCr and < 30 mg/gCr by the uPCR, Probability (uPCR, uCr), and Probability (uPCR, uSG) in non-diabetic patients with G stages 1 to 3a and dipstick -negative proteinuria by the ROC curve. **c** Differentiation of uACR ≥ 30 mg/gCr and < 30 mg/gCr by the uPCR, Probability (uPCR, uCr), and Probability (uPCR, uSG) in non-diabetic patients with G stage 3b to 4 and dipstick-negative proteinuria by the ROC curve. The results are shown in Supplemental Table 11

Figure 4a-c and Supplemental Table 11 indicate that while the probability of predicting uACR ≥ 30 mg/gCr based on a logistic model using uPCR and uCr or uPCR and urine specific gravity (uSG) in negative proteinuria for non-diabetic patients was significantly more useful in differentiating uACR ≥ 30 mg/gCr in G1-4 and G1-3a, than uPCR alone, there was no marked difference in G3b-4. The differentiation of uACR ≥ 30 mg/gCr by the predictive probability based on a logistic model using the uPCR and uCr or the uPCR and uSG in diabetic patients with negative proteinuria was significantly higher in G1-4 than the differentiation using uPCR alone.

Supplemental Fig. 8a,b, and Supplemental Table 12 show that, in non-diabetic and diabetic patients with negative proteinuria, there was a significant negative correlation between the uCr or uSG and the uACR at A1L, A1M, and A2.

**Correlation between the changes in uPCR and changes in uACR **(Supplemental Fig. 9 a, b, Supplemental Table 13).

The Supplemental Table 13 indicates the correlation between the changes in uPCR and changes in uACR. Even though the urinary protein was negative in all three times, the changes in both were significantly positively correlated, and the increase/decrease in uACR per uPCR 0.01 g/gCr was approximately 3 mg/gCr in non-diabetic and diabetic patients. When urinary protein qualitative findings were trace or more, the increase/decrease in uACR per 0.01 g/gCr of uPCR increased to approximately 5–6 mg/gCr in non-diabetic and diabetic patients.

## Discussion

Regarding the detection of uACR ≥ 30 mg/gCr by a single dipstick measurement, the sensitivity + specificity—1 value was the highest in non-diabetic and diabetic patients with more than trace proteinuria, as shown in Supplemental Table 2. Findings in previous reports have varied, reporting values of “sensitivity 37.1%, specificity 97.3%, positive predictive value (PPV) 71.4%, negative predictive value (NPV) 89.5%” [11]; “sensitivity 69.4%, specificity 86.8%, PPV 27.1%, NPV 97.6%” [12]; and “sensitivity 43.6% specificity 93.6%, PPV 34.6%, NPV 95.5%” [13] for more than trace proteinuria. This difference is believed to be due to the fact that there are many cases of advanced CKD, as the subjects in this study were outpatients with lifestyle-related diseases, and because dipstick evaluations are affected by the urine concentration and test reagents [20]. Although it is necessary to confirm the results when the PPV is low, even if the sensitivity of uACR ≥ 30 mg/gCr is high [12, 13], this study indicated that the PPV was high and suggested the utility of dipstick test, even if a dipstick proteinuria evaluation showed low sensitivity. However, as the NPV was relatively low, a uACR of ≥ 30 mg/gCr cannot be ruled out in cases of negative proteinuria.

When the utility of a single dipstick evaluation was compared with that of the uPCR for detecting of uACR ≥ 30 mg/gCr, the uPCR was shown to be more useful, with a high sensitivity and NPV as well as an almost equivalent specificity and PPV.

It is recommended that microalbuminuria be diagnosed, even when a single uACR measurement is ≥ 30 mg/gCr, if another examination shows uACR ≥ 30 mg/gCr among 2 subsequent early-morning urine tests [3]. Regarding the significance of the three-time dipstick measurement, if the CO of 1 measurement is trace proteinuria, 3 measurements are more useful than 1 measurement, with the CO of 3 measurements indicating sensitivity 55.5% and specificity 79.7% when trace or more proteinuria is shown even once out of 3 times. If the CO of microalbuminuria is set at trace or more proteinuria two out of three times or 1( +) or more proteinuria one out of three times, the sensitivity for detecting microalbuminuria is reduced in non-diabetic patients, and therefore when the dipstick test shows trace levels or higher proteinuria once, it is desirable to confirm the result by measuring either uPCR or uACR at that time.

The sum of three uPCRs in non-diabetic patients was useful for differentiating microalbuminuria and indicated a CO (D, YI) of 0.23 g/gCr, which is approximately 3 times the CO of a single uPCR measurement. However, for qualitative proteinuria measurements, the CO (D, YI) was set at a trace finding being obtained at least one out of three times. The reason for this may be because the median uACRs of trace proteinuria in non-diabetic and diabetic patients were 56 and 73 mg/gCr, respectively, while the first quartiles were at 32.5 and 42 mg/gCr, respectively, which were higher than 30 mg/gCr.

The uPCR was useful for differentiating between uACR < 30 mg/dl and ≥ 30 mg/dl, when we limited our studies to dipstick-negative proteinuria. Furthermore, a single uPCR measurement was useful for predicting microalbuminuria, even when evaluating proteinuria-negative cases via the dipstick test three times in a row, with the sum of three uPCRs also being useful. While proteinuria is generally quantified after detection of proteinuria by a dipstick examination [8], measuring the uPCR, even wnen the result is negative, seems useful for the early detection of microalbuminuria in lifestyle-related diseases.

The CO (YI) of uPCR that differentiates uACR ≥ 30 mg/dl in non-diabetic lifestyle-related diseases remained unchanged from the CO of negative proteinuria, with the values for G1-3a and G3b-4 being 0.07 g/gCr and 0.10 g/gCr, respectively, even when more than trace proteinuria was included. The sensitivity at the same uPCR and AUC increased while the specificity showed a small decrease when more than trace proteinuria was included. This is believed to be due to the fact that the COs is in A1M, while the uACR is mostly ≥ 30 mg/gCr in A1H and A2, along with the fact that the high fraction ratio of uPCR increases with the increase in qualitative proteinuria findings.

Regarding the difference in CO (YI) between G1-3a and 3b-4, the specificity of G1-3a increased more rapidly with the increase in the CO of the uPCR than did that of G3b-4, whereas G3b-4 had a slower decrease in sensitivity than G1-3a. This difference is attributed to the significant difference in the number of cases with uACR < 30 mg/gCr and ≥ 30 mg/gCr by uPCR category between G1-3a and G3b-4, meaning that G1-3a had higher proteinuria selectivity than G3b-4 or tubular protein in the urine may be increased in G3b-4.

The predictive probability of uACR ≥ 30 mg/gCr based on a logistic model using uPCR and the uSG or uCr in both non-diabetic and diabetic patients with negative proteinuria was significantly better than the uPCR alone for differentiating of uACR ≥ 30 mg/gCr. This appears to be due to the fact that the uSG, uCr and uACR exhibit a negative correlation at the same uPCR value of ≤ 0.10 g/gCr, suggesting that the uACR may be increased in diluted urine and decreased in concentrated urine. While the reason for this is unclear, the reabsorption of filtered albumin in the renal proximal tubules [21] may be increased in concentrated urine compared with nonalbumin protein in the urine.

The KDIGO CKD guideline [3] recommended that even if a uACR ≥ 30 mg/gCr is noted in a single spot urine measurement, the results should be confirmed by measuring the uACR using early-morning urine in order to exclude the possibility of postural proteinuria. Another reason for using the early morning urine based on the findings of this study is that the uACR of the specimen may have been reduced, as early-morning urine is expected to be more concentrated than spot urine. When predicting the uACR from the uPCR, it may also be desirable to use early-morning urine, although the uPCR itself is a major predictive factor of microalbuminuria.

The uACR estimates the daily albuminuria excretion under the assumption that the daily urinary creatinine excretion is 1 g, with a good correlation reportedly having been shown in patients with a normal renal function [22]. It appears that the significance thereof in G3b and G4 is unclear. The urinary Cr excretion per unit time is expressed as (serum Cr value × GFR + amount of creatinine secreted by renal tubules). As renal dysfunction progresses, the tubular secretion of creatinine per unit nephron increases, while the number of functional nephrons is expected to decrease. While serum creatinine increases as eGFR decreases, this study found that the median age at stages G1, 2, 3a, 3b and 4 indicated 46.4, 60.6, 71.0, 77.3 and 79.5 years old for non-diabetic patients, respectively, and 55.0, 65.7, 75.0, 76.1 and 76.0 years old for diabetic patients, respectively, with significantly more elderly patients showed a decreased renal function than younger patients (Kruskal–Wallis test *P* < 0.001, respectively). In addition to aging, a study on CKD patients with minimal dietary interventions reported that patients with lower creatinine clearance had a spontaneous decrease in dietary protein intake, reduced 24-h urine Cr excretion [23], and prevalence of sarcopenia is increased as CKD progressed [24]. Judging from the above, it is predicted that muscle mass will decrease in patients with a decreased renal function, with serum Cr value × GFR also potentially decreasing. With respect to improving the limitations of the daily albuminuria excretion prediction by uACR, it has been reported that the estimated albumin excretion rate, calculated by multiplying the spot uACR value by the estimated urinary creatinine excretion rate (g/24 h), improved the prediction of the measured 24-h albumin excretion [25]. Nonalbuminuric renal dysfunction have been reported in type 2 diabetic patients and the general population [26, 27], so it is necessary to investigate the estimated albumin excretion rate and the estimated protein excretion rate for predicting it in cases of a decreased renal function.

The BMI and abdominal circumference were higher in diabetic patients than in non-diabetic patients, and while significant positive correlations between the BMI and uPCR and the BMI and uACR were noted in non-diabetic patients, no such correlations were noted in diabetic patients. The lack of correlations in diabetic patients may be due to a small number of this study. It is reported that high waist-to-hip ratio and BMI are independently positively associated with albuminuria due to intraglomerular haemodynamics resulting from excess adiposity [28]. uACR and uPCR were higher in hypertensive cases than in non-hypertensive cases among non-diabetic patients in this study. There is the possibility that hypertension and obesity correlate with the severity of global nephrosclerosis in non-diabetic nephrosclerosis [29], and it is speculated that systemic and intraglomerular hypertension cause glomerular sclerosis, resulting in high uACR and uPCR values in non-diabetic hypertensive or obese patients. In addition, glomerular lesions, which are typically found in diabetic nephropathy (diabetic glomerulopathy) [29], may be involved in the increase in uACR and uPCR in diabetic patients.

In this study, uPCR was useful for determining microalbuminuria in both non-diabetic and diabetic patients, even when testing negative using a dipstick test, and the CO of uPCR for microalbuminuria in cases with three consecutive negative proteinuria findings for both groups was 0.06 g/gCr. In Japan, the measurement of albuminuria is covered by insurance only when incipient diabetic nephropathy is supposed. Obesity [30] and metabolic syndrome [31] are risk factors for diabetes. uACR and uPCR were high in obese and hypertensive cases of non-diabetic patients in this study. Therefore, in the group at high-risk for diabetes, even if the patient is found to be a proteinuria-negative case based on the findings of a dipstick test, it is desirable to measure uPCR, and by maintaining uPCR at a level of ≤ 0.05 g/gCr, it is possible for the albuminuria to remain in the normal range, even if diabetes has already developed. Should microalbuminuria be suspected, then encouraging the patient to make changes in their lifestyle may reduce albuminuria, decrease the risk of a progression to diabetes, and also prevent the development of nephrosclerotic lesions in cases that have developed diabetes.

Albuminuria is a risk factor for renal dysfuction and cardiovascular disease in diabetic patients [4], and this study targeted patients with normo- and microalbuminuria. Exacerbation of albuminuria indicates an increasing risk of renal dysfunction and cardiovascular disease, while amelioration from macroalbuminuria to microalbuminuria or microalbuminuria to normoalbuminuria indicates a reduced risk following the onset of diabetes [4, 6, 32]. This study indicated that changes in uACR and uPCR exhibited a significant positive correlation even if urinary protein was negative three times via the dipstick test, with changes in uACR appearing to be predictable from changes in uPCR. When the dipstick proteinuria was negative three times, the increase/decrease in uACR per uPCR 0.01 g/gCr was approximately 3 mg/gCr, which tended to be lower than approximately 5–6 mg/gCr which included trace or more urinary protein qualitative findings. This is probably because uPCR values in cases of positive dipstick protein findings are higher than the uPCR values in cases of negative proteinuria, and the ratio of albumin in total proteinuria increases as uPCR increases. In Japan, uACR measurement is covered by insurance only once in three months for diabetic patients, so uPCR may be able to evaluate the therapeutic effect and detect deterioration at an early stage during that period.

It has been reported that the use of RAAi (such as ACEi, ARB, and MRB) [10, 33, 34] and SGLT2i [10, 35] reduces albuminuria and the accompanying renal dysfunction and cardiovascular disease in patients with chronic kidney disease with or without diabetic patients. Although the use of ACEi and ARB has been reported to be associated with high uACR/uPCR levels [36], the effects of SGLT2i are unclear. This study indicated that the CO of uPCR, which distinguishes uACR 30 mg/gCr or more in cases using SGLT2i, tended to be higher than that in non-use cases (Supplemental Table 14), so the effect of taking SGLT2i on uACR/uPCR and the CO of microalbuminuria shall be investigated going forward.

The inconsistency between the CO (D) and CO (YI) was recognized in differentiating uACR ≥ 30 mg/gCr in proteinuria-negative cases among non-diabetic patients with G3b-4 and microalbuminuria in triple proteinuria-negative cases of G1-3a. The CO (YI) is reportedly preferable because it maximizes the overall rate of correct classification when the criteria do not agree [19]. However, D^2^ = min [-2(sensitivity + specificity -1) + (sensitivity^2^ + specificity^2^)], and (sensitivity^2^ + specificity^2^) is smallest at the CO where the sensitivity and specificity are equal and when (sensitivity + specificity) is constant. In cases of lifestyle-related diseases, the risk of cardiovascular disease increases starting at uACR < 30 mg/gCr [5], so there is little disadvantage in intervening in patients with normoalbuminuria. As high sensitivity for detecting microalbuminuria is considered　necessary for early prevention of cardiovascular disease, the CO (D) seems useful in that it takes into consideration both the accuracy and the balance between sensitivity and specificity.

One limitation associated with this study was its single-center setting, with different results potentially being obtained depending on the target patient, dipstick test, proteinuria, albumin quantification measurement conditions, and measurement methods [20]. In addition, the number of patients was relatively small, but there have been no reports evaluating the factors for microalbuminuria based on urine dipstick, uPCR, and uACR measurements in the same urine sample three times. The median time between the three uACR measurements was about four to five months, which was relatively long, so the results may have been affected by the treatment of lifestyle-related diseases during this period, although the KDIGO guideline recommends a further two measurements of the uACR within two months after the first measurement [3].

The main results of this study were summarized in Table 5.

**Table 5:** Summary of prediction of microalbuminuria from urine dipstick and uPCR in non-diabetic and diabetic life-style related disease

prediction of microalbuminuria	
urine dipstick	A one-time dipstick proteinuria examination is useful for predicting microalubuminuria, with uPCR or uACR having to be measured and confirmed at ≥ trace( ±)
	Three-time dipstick proteinuria examinations are more useful for predicting microalubuminuria than the one-time dipstick examination, with uPCR or uACR having to be measured and confirmed upon the first ≥ trace( ±)
uPCR	Among all proteinuria findings on urine dipsticks, uPCR is useful for predicting microalbuminuria
	Even if the urinary protein tests negative using a urine dipstick, measuring uPCR is useful for the early detection of microalbuminuria
	By maintaining the value of the one-time uPCR at ≤ 0.05 g/gCr^a^, as well as the sum of the three-time uPCRs at ≤ 0.22 g/gCr^b^, it may be possible to maintain normoalbuminuria
	It may be microalbuminuria if the one-time uPCR is ≥ 0.06 g/gCr or the sum of the three-time uPCRs is ≥ 0.23 g/gCr, requiring intervention such as confirmation by measuring uACR or further improvement in lifestyle
prediction of change in uACR	
	Among all proteinuria findings on urine dipsticks, including dipstick proteinuria negative, an increase or decrease in uACR can be predicted by an increase or decrease in uPCR
sample	A spot first-morning urine sample is desirable, which is relatively concentrated and is not influenced by postural proteinuria

*uPCR* urine protein-to-creatinine ratio, *uACR* urine albumin-to-creatinine ratio

Normoalbuminuria: uACR < 30 mg/gCr at least twice for 3 measurements

Microalbuminuria: uACR 30–299 mg/gCr at least twice for 3 measurements

^a^2nd uPCR

^b^Sum of the 1st, 2nd and 3rd uPCRs

## Conclusions

While a urine dipstick evaluation is useful for detecting microalbuminuria in cases of non-diabetic lifestyle-related diseases, measuring the uPCR, preferably using the early-morning urine three times, including in dipstick-negative proteinuria cases, may lead to the early detection of microalbuminuria and prompt intervention for CKD due to non-diabetic lifestyle-related disease.

## Supplementary Information

**Additional file 1: Supplemental Figure 1.** **a** The association between the sum of the three urine protein dipstick scores (SuPDS) and the sum of the three uPCRs in non-diabetic patients. The sum of the three uPCRs (g/gCr) = 0.2173 × SuPDS + 0.1905, R=0.759, P=5.3×10E-60. **b** The association between the sum of the three urine protein dipstick scores (SuPDS) and the sum of the three uPCRs in diabetic patients. The sum of the three uPCRs (g/gCr) = 0.1771 × SuPDS + 0.2525, R=0.591, P=1.1×10E-13. **Supplemental Figure 2.** **a** The ROC curve for the differentiation of uACR ≥30 mg/gCr in non-diabetic patients with G stage 3b to 4 and dipstick proteinuria(-). AB: minimum distance, BC: maximal (1 - Distance), EG: maximal (Se + Sp - 1) at Youden’s index. The association between CO of the uPCR and sensitivity, specificity, Se + Sp - 1, and 1 - Distance when turning from (1 - specificity: 1, sensitivity: 1) to (1 - specificity: 0, sensitivity: 0) clockwise around (1 - specificity: 0, sensitivity: 1) is shown in (b). The specificity of point B is lower than that of point E, but has a higher sensitivity. **b** The association between the CO of the uPCR and the sensitivity, specificity, Se+Sp-1, and 1 - Distance for the differentiation of uACR <30 mg/gCr and ≥30 mg/gCr in non-diabetic patients with stage G 3b to 4 and dipstick proteinuria (-). The sensitivity, specificity, Se+Sp-1, 1-Distance at Point B where the distance of the ROC curve was the smallest, corresponds to the sensitivity, specificity, Se+Sp-1, 1-Distance at uPCR 0.07 g/gCr in (b). The sensitivity, specificity, Se+Sp-1, 1-Distance at Point E of Youden's Index on the ROC curve corresponded to the sensitivity, specificity, Se+Sp-1, and 1-Distance at uPCR 0.10g/gCr in (b). Although the inconsistency between optimal CO by maximal (1-Distance) and YI was recognized in this example, the CO of uPCR at B can be regarded as the optimal CO, since the Se+Sp-1 of BD and EG are nearly equal and B is more sensitive than E. The association between the CO and accuracy is clear in this graph, and it is possible to create an ROC curve from this graph as well. **Supplemental Figure 3.** **a** The distribution of proteinuria by urine dipstick according to the uPCR and uACR in non-diabetic patients with G stages 1 to 4. ○: proteinuria(-), □: proteinuria(±), △: proteinuria(+), ▽: proteinuria 2(+), ◇: proteinuria 3(+). The associations between the uPCR and median uACR are shown by a restricted cubic spline using 4 knots. Adjusted R-squared = 0.959, Akaike’s information criterion (AIC) = -99.0. The 4 knots were at percentiles 5, 35, 65, and 95 of all measurements. The two vertical lines indicate a uPCR of 150 and 500 mg/gCr, while the two horizontal lines indicate a uACR of 30 and 300 mg/gCr. The restricted cubic spline shows that a uACR of 30 mg corresponds to a uPCR of 0.076 g/gCr, and a uPCR of 0.15 g/gCr corresponds to a uACR of 80 mg/gCr. **b** The distribution of proteinuria by urine dipstick according to the uPCR and uACR in diabetic patients with G stage 1 to 4. ○: proteinuria(-), □: proteinuria(±), △: proteinuria(+), ▽: proteinuria 2(+). The associations between the uPCR and median uACR are shown by a restricted cubic spline using 4 knots. Adjusted R-squared = 0.940, AIC = -67.2. The two vertical lines indicate a uPCR of 150 and 500 mg/gCr, while the two horizontal lines indicate a uACR of 30 and 300 mg/gCr. The restricted cubic spline shows that a uACR of 30 mg/gCr corresponds to a uPCR of 0.073 g/gCr, and a uPCR of 0.15 g/gCr corresponds to a uACR of 73 mg/gCr. **Supplemental Figure 4.** **a** The ROC curve for the differentiation of uACR ≥30 mg/gCr based on the urine protein dipstick score and uPCR in non-diabetic patients with G stages 1 to 4. The uPCR was more useful for differentiating uACR ≥30 mg/gCr than a dipstick measurement in non-diabetic patients (p<0.0001). **b** The ROC curve for the differentiation of uACR ≥30 mg/gCr based on the urine protein dipstick score and uPCR in diabetic patients with G stages 1 to 4. The uPCR was more useful for differentiating uACR ≥30 mg/gCr than a dipstick measurement in diabetic patients (p<0.0001). **Supplemental Figure 5.** **a** The utility of the cut-off values of the uPCR and sensitivity (Se), specificity (Sp), Se+Sp-1, and 1-Distance in the differentiation of uACR ≥30 mg/gCr in non-diabetic patients with G stages 1 to 4 according to dipstick proteinuria. Red: patients with proteinuria(-), Green: patients with proteinuria(-)(±), Blue: patients with proteinuria(-)(±)(+), Purple: patients with proteinuria(-)(±)(+)2,3(+), ○:Sensitivity, □: Specificity, △: Se+Sp-1, ▽: 1 - Distance. The solid vertical line indicates CO (YI), uPCR 0.07 g/gCr at dipstick proteinuria(-) and (-)(±) and uPCR 0.09 g/gCr at dipstick proteinuria(-)(±)(+) and (-)(±)(+)2,3(+). The results are shown in Table 2. **b** The association of the CO of the uPCR and ratio of sensitivity and specificity versus dipstick proteinuria(-) in the differentiation of uACR ≥30 mg/gCr in non-diabetic patients with G stages 1 to 4. Ratio of sensitivity in patients with dipstick proteinuria (-)(±)(●Ratio Se1), (-)(±)(+) (■Ratio Se2), and (-)(±)(+),2.3(+)(▲Ratio Se3) versus patients with dipstick proteinuria (-). Ratio of specificity in patients with dipstick proteinuria(-)(±)(○Ratio Sp1), (-)(±)(+)(□Ratio Sp2), and (-)(±)(+),2,3(+)(△Ratio Sp3) versus patients with dipstick proteinuria(-).While the ratio of sensitivity increases with an increase in the CO of uPCR, the CO of uPCR with the largest Se+Sp-1 and 1 - Distance, exists in A1M (uPCR 0.06-0.10g / gCr). The ratio of specificity hardly changed with the increase in the uPCR. **Supplemental Figure 6.** **a**, **b** The association between the cut-off values of the uPCR and differences in the sensitivity and specificity for predicting uACR ≥30 mg/gCr between G stage 1-3a and G stage 3b-4 in patients with non-diabetic lifestyle-related diseases according to dipstick proteinuria. Although the specificity of G1-3a was higher than that of G3b-4 at uPCR 0.07 g/gCr [the CO(YI) of G1-3a) ], the sensitivity of G1-3a was lower than that of G3b-4 at the CO. Although the sensitivity of G3b-4 was higher than that of G1-3a at uPCR 0.10 g/gCr [the CO(YI) of G3-4) ], the specificity of G3b-4 was lower than that of G1-3a at the CO. **Supplemental Figure 7.** Association between logit p and probability of microalbuminuria. Probability [1,2,3 dipstick≥(+)]: probability of micro- and macroalbuminuria by a logistic model using the 1st, 2nd and 3rd dipstick urine protein. Dummy variable dipstick proteinuria≥(+);1 and (±)(-); 0, respectively. Probability[1,2,3 dipstick≥(±)] : probability of micro- and macroalbuminuria by a logistic model using the 1st, 2nd and 3rd dipstick urine protein. Dummy variable dipstick proteinuria≥(±);1 and (-); 0, respectively.　●: Cut-off value (D, YI) of probability[1,2,3 dipstick≥(+)] for predicting microalbuminuria or worse. ■: Cut-off value (D, YI) of Probability[1,2,3 dipstick≥(±)] predicting microalbuminuria or worse. In the case of dipstick urine protein ≥ 1 (+): 1, (±), (-): 0, the determination coefficient was 0.109 and the association between logit p and probability of microalbuminuria in the logistics model fluctuated from (logit p 0.620, probability 0.650) to (4.512, 0.989), except when the urinary protein was negative in all three times (model A). In the case of dipstick urine protein ≥1 (±): 1, (-): 0, the determination coefficient was 0.135, the association between logit p and probability of microalbuminuria in the logistics model fluctuates from (logit p 0.129, probability 0.532) to (2.420, 0.918) , except when the urinary protein was negative in all three times, and the sensitivity and accuracy in CO for detecting microalbuminuria were higher (model B). The correlation between the three urine examination findings of Model B and the accuracy of detection of microalbuminuria is shown in Supplemental Table 5. **Supplemental Figure 8.** **a** The association between the urine creatinine level and uACR in non-diabetic patients with dipstick-negative proteinuria according to the uPCR. **b** The association between the urine specific gravity and the uACR in non-diabetic patients with dipstick-negative proteinuria according to the uPCR. A1L: uPCR 0-0.05 g/gCr, A1M: uPCR 0.06-0.10 g/gCr, A1H: uPCR 0.11-0.14 g/gCr, A2: uPCR 0.15-0.49 g/gCr, A3: uPCR≥0.50 g/gCr. The median uACR increases along with the the increase in A stage by the uPCR. In contrast, the uCr and uSG showed a negative correlation with the uACR for each A stage. These results indicate that the uPCR and uCr or uSG were independently associated with the uACR in non-diabetic patients with dipstick-negative proteinuria.The results are shown in Supplemental Table 12. **Supplemental Figure 9.** **a** Correlation between the changes in uPCR (second uPCR - first uPCR) and changes in uACR (second uACR - first uACR) in diabetic patients. ●：SuPDS 0, ○：SuPDS 0.5-4.0. **b** Correlation between the changes in uPCR(third uPCR - second uPCR) and changes in uACR(third uACR - second uACR) in diabetic patients. ●：SuPDS 0, ○：SuPDS 0.5-4.0. The results are shown in Supplemental Table 13.

**Additional file 2: Supplemental Table 1.** A comparison of laboratory data according to the results of the dipstick proteinuria in non-diabetic patients. **Supplemental Table 2.** Detection of uACR ≥30 mg/gCr by dipstick proteinuria according to G stage in non-diabetic and diabetic patients. **Supplemental Table 3.** A comparison of the ability to predict microalbuminuria or worse using the dipstick proteinuria, uPCR, and uACR in diabetic patients with G1-4 and all qualitative proteinuria findings. **Supplemental Table 4.** Prediction of microalbuminuria or worse by a logistic model using three consecutive dipstick proteinuria findings in non-diabetic patients. **Supplemental Table 5.** Detection of microalbuminuria or worse based on three consecutive dipstick proteinuria findings in non-diabetic patients. **Supplemental Table 6.** Prediction of microalbuminuria or worse by a logistic model based on three consecutive uPCRs in non-diabetic and diabetic patients. **Supplemental Table 7.** Prediction of microalbuminuria or worse based on a single second uPCR value according to the G stage and the sum of three urine protein dipstick scores in non-diabetic and diabetic patients. **Supplemental Table 8.** A comparison of the chracteristics between cases of uACR<30 mg/gCr and ≥30 mg/gCr in non-diabetic and diabetic patients with dipstick-negative proteinuria. **Supplemental Table 9.** Factors associated with uACR ≥30 mg/gCr in non-diabetic patients with dipstick-negative proteinuria. **Supplemental Table 10.** Factors associated with uACR ≥30 mg/gCr in non-diabetic and diabetic patients with dipstick-negative proteinuria. **Supplemental Table 11.** Differentiation of uACR ≥30 mg/gCr and <30 mg/gCr by uPCR, probability of uACR ≥30 mg/gCr based on a logistic model using uPCR and urine creatinine or urine specific gravity according to the G stage in non-diabetic and diabetic patients with dipstick-negative proteinuria. **Supplemental Table 12.** Associations between urine creatinine or specific gravity and urinary albumin-to-creatinine ratio according to the urine protein-to-creatinine ratio in non-diabetic and diabetic patients with dipstick-negative proteinuria. **Supplemental Table 13.** Association between changes in the uPCR and in the uACR among non-diabetic and diabetic patients. **Supplemental Table 14.** The effect of medication on the CO for uPCR for differentiating diabetic patients with uACR ≥30 and <30 mg/gCr.

## Data Availability

The datasets used and analysed during the current study are available from the corresponding author on reasonable request.

## Abbreviations

CKD: Chronic kidney disease
KDIGO: Kidney disease improving global outcomes
BMI: Body mass index
uPCR: Urine protein-to-creatinine ratio
uACR: Urine albumin-to-creatinine ratio
eGFR: Estimated glomerular filtration rate
SuPDS: Sum of the urine protein dipstick score
uSG: Urine specific gravity
uCr: Urine creatinine
ASO: Antistreptolysin-O
MPO-ANCA: Myeroperoxidase antineutrophil cytoplasmic antibody
PR3-ANCA: Proteinase3 antineutrophil cytoplasmic antibody
NAG: N-acetyl-β-D-glucosaminidase
L-FABP: Liver-type fatty acid-binding protein
ROC: Receiver operating characteristic
AUC: Area under the curve
SE: Standard error
CI: Confidence interval
CO: Cut-off value
YI: Youden's Index
D: Distance
Se: Sensitivity
Sp: Specificity
PPV: Positive predictive value
NPV: Negative predictive value
RAAi: Renin–angiotensin–aldosterone system inhibitor
ACEi: Angiotensin-converting enzyme inhibitor
ARB: Angiotensin II receptor blocker
MRB: Mineralocorticoid receptor blocker
