# Supplementary material for: A comparison of the utility of the urine dipstick and urine protein-to-creatinine ratio for predicting microalbuminuria in patients with non-diabetic lifestyle-related diseases -a comparison with diabetes
Source: BMC Nephrol. 2022 Nov 24;23:377. doi: 10.1186/s12882-022-02974-6 (PMC9700904; doi:10.1186/s12882-022-02974-6)
Supplement: Supplementary file 1 — Additional file 1: Supplemental Figure 1. a The association between the sum of the three urine protein dipstick scores (SuPDS) and the sum of the three uPCRs in non-diabetic patients. The sum of the three uPCRs (g/gCr) = 0.2173 × SuPDS + 0.1905, R=0.759, P=5.3×10E-60. b The association between the sum of the three urine protein dipstick scores (SuPDS) and the sum of the three uPCRs in diabetic patients. The sum of the three uPCRs (g/gCr) = 0.1771 × SuPDS + 0.2525, R=0.591, P=1.1×10E-13. Supplemental Figure 2. a The ROC curve for the differentiation of uACR ≥30 mg/gCr in non-diabetic patients with G stage 3b to 4 and dipstick proteinuria(-). AB: minimum distance, BC: maximal (1 - Distance), EG: maximal (Se + Sp - 1) at Youden’s index. The association between CO of the uPCR and sensitivity, specificity, Se + Sp - 1, and 1 - Distance when turning from (1 - specificity: 1, sensitivity: 1) to (1 - specificity: 0, sensitivity: 0) clockwise around (1 - specificity: 0, sensitivity: 1) is shown in (b). The specificity of point B is lower than that of point E, but has a higher sensitivity. b The association between the CO of the uPCR and the sensitivity, specificity, Se+Sp-1, and 1 - Distance for the differentiation of uACR <30 mg/gCr and ≥30 mg/gCr in non-diabetic patients with stage G 3b to 4 and dipstick proteinuria (-). The sensitivity, specificity, Se+Sp-1, 1-Distance at Point B where the distance of the ROC curve was the smallest, corresponds to the sensitivity, specificity, Se+Sp-1, 1-Distance at uPCR 0.07 g/gCr in (b). The sensitivity, specificity, Se+Sp-1, 1-Distance at Point E of Youden's Index on the ROC curve corresponded to the sensitivity, specificity, Se+Sp-1, and 1-Distance at uPCR 0.10g/gCr in (b). Although the inconsistency between optimal CO by maximal (1-Distance) and YI was recognized in this example, the CO of uPCR at B can be regarded as the optimal CO, since the Se+Sp-1 of BD and EG are nearly equal and B is more sensitive than E. The association betwe [file 12882_2022_2974_MOESM1_ESM.pptx]

## Slide 1
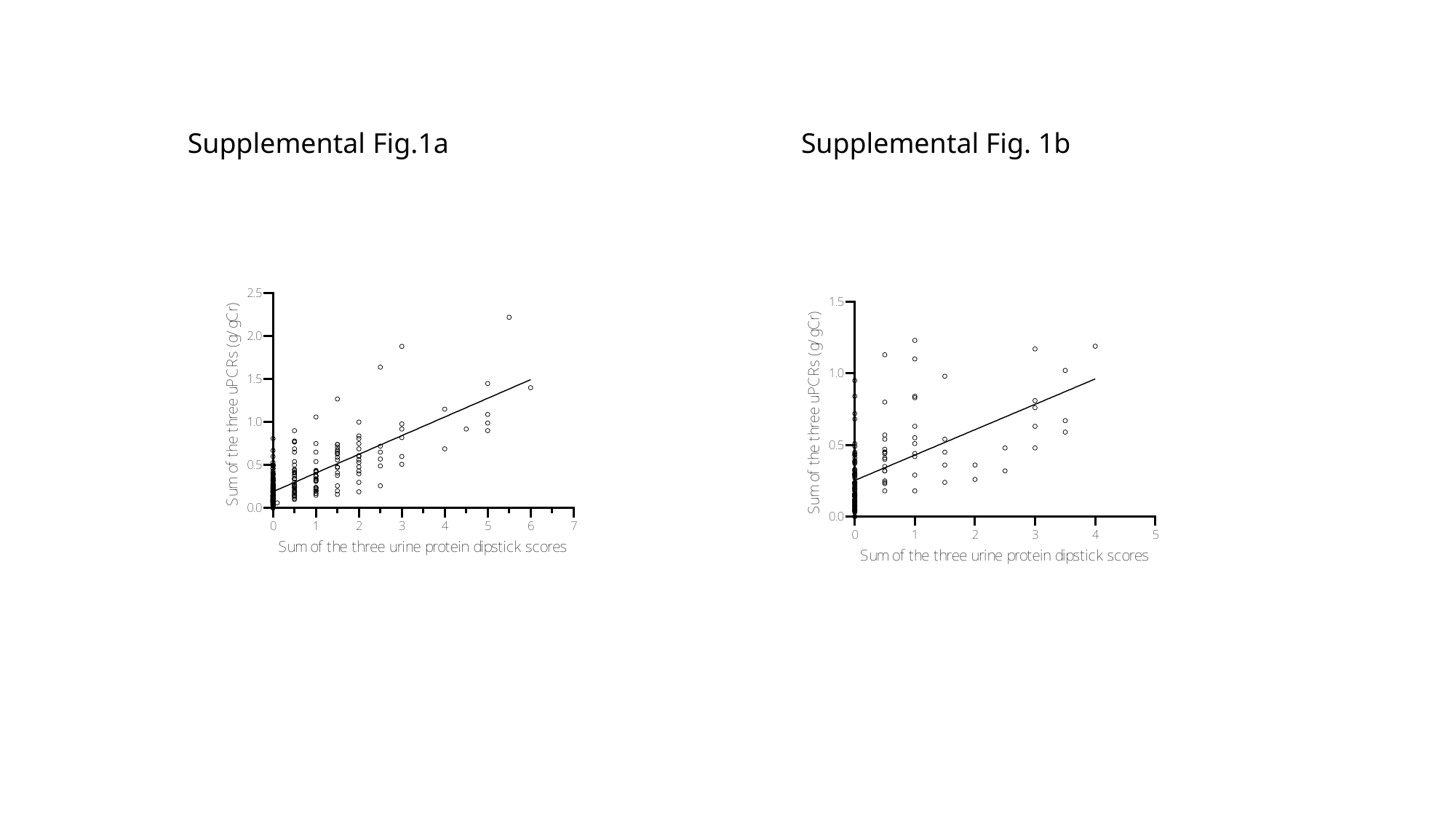

Supplemental Fig.1a
Supplemental Fig. 1b

## Slide 2
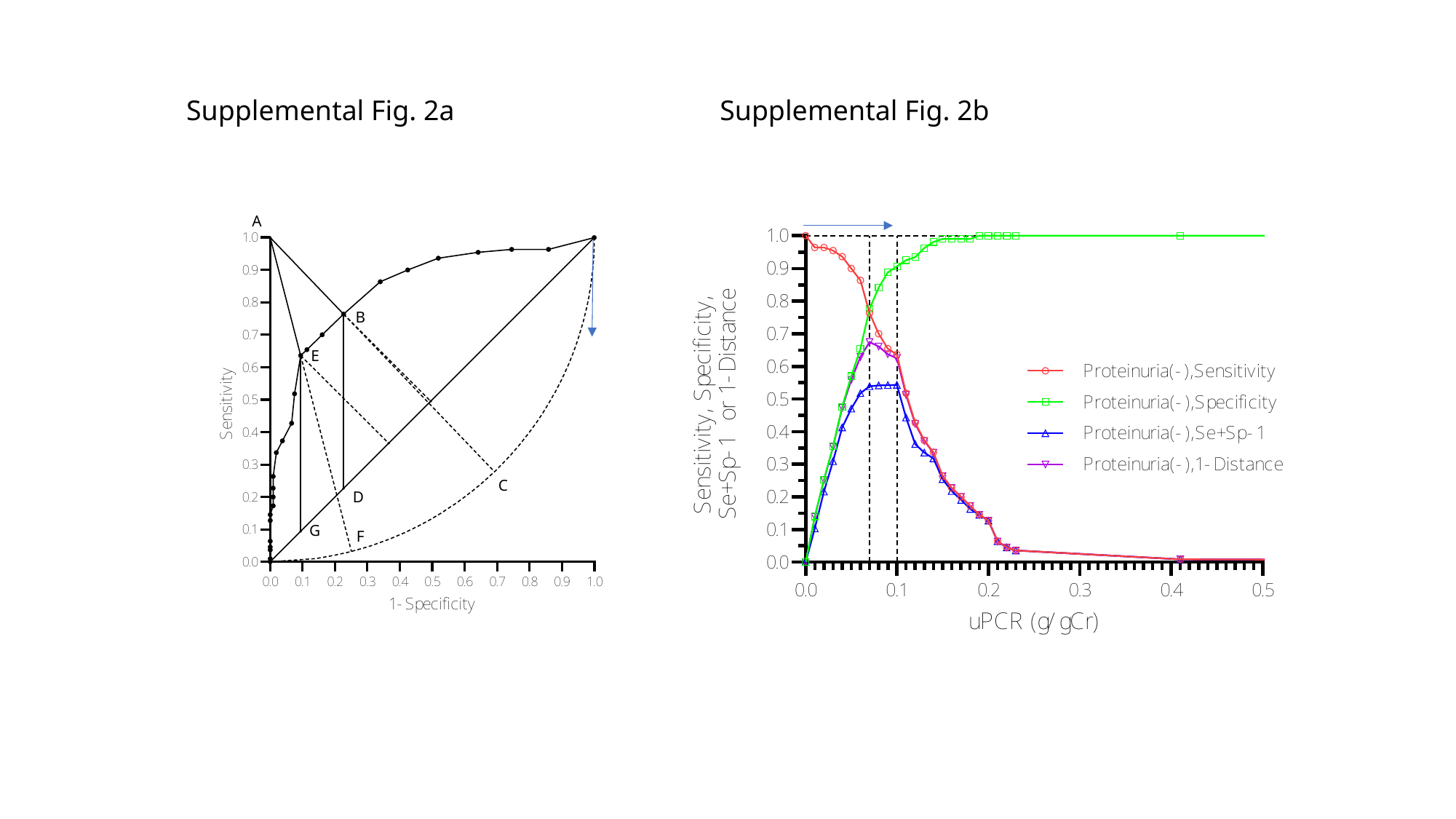

Supplemental Fig. 2a
Supplemental Fig. 2b
A
B
E
C
D
G
F

## Slide 3
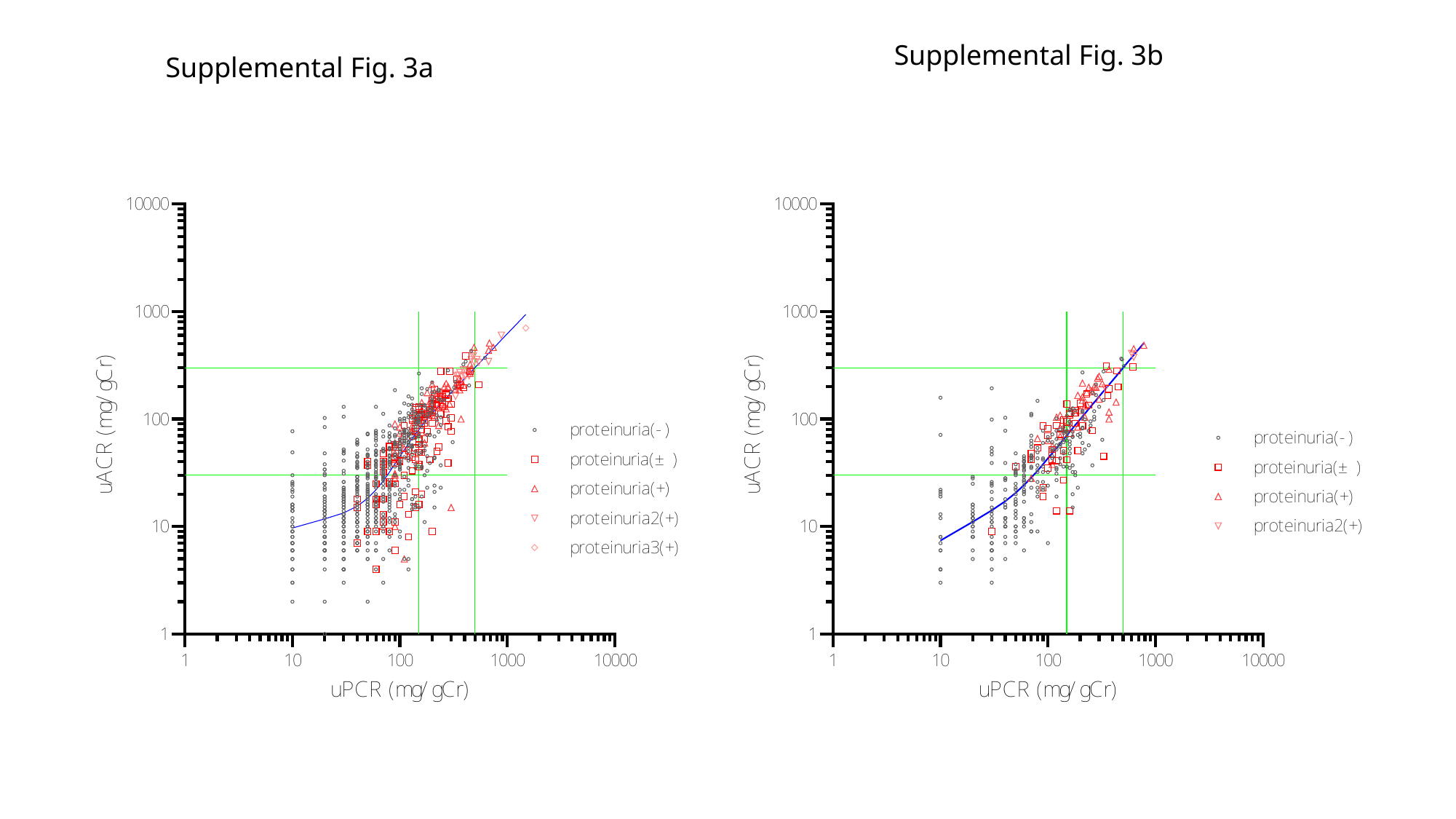

Supplemental Fig. 3b
Supplemental Fig. 3a

## Slide 4
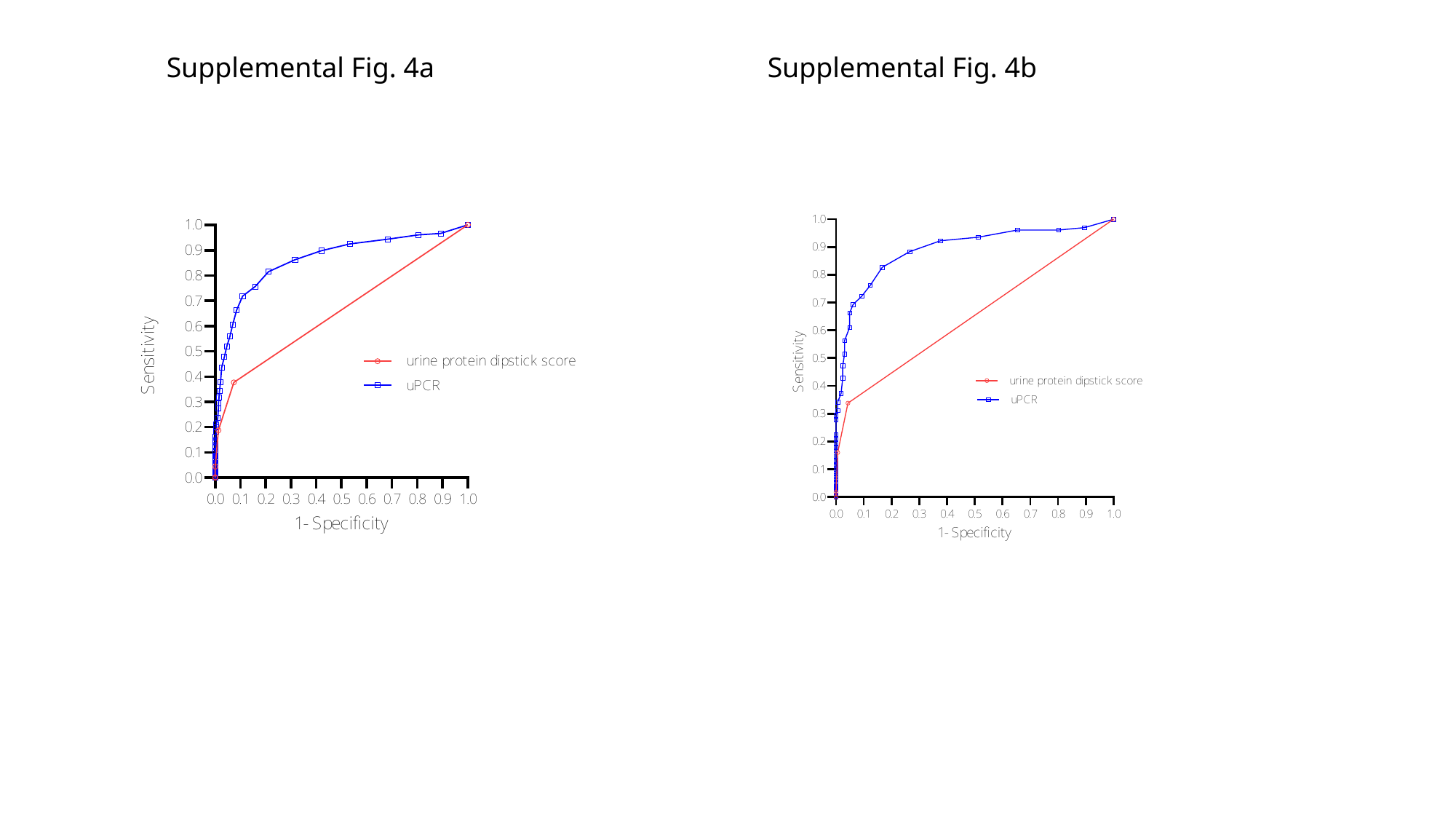

Supplemental Fig. 4a
Supplemental Fig. 4b

## Slide 5
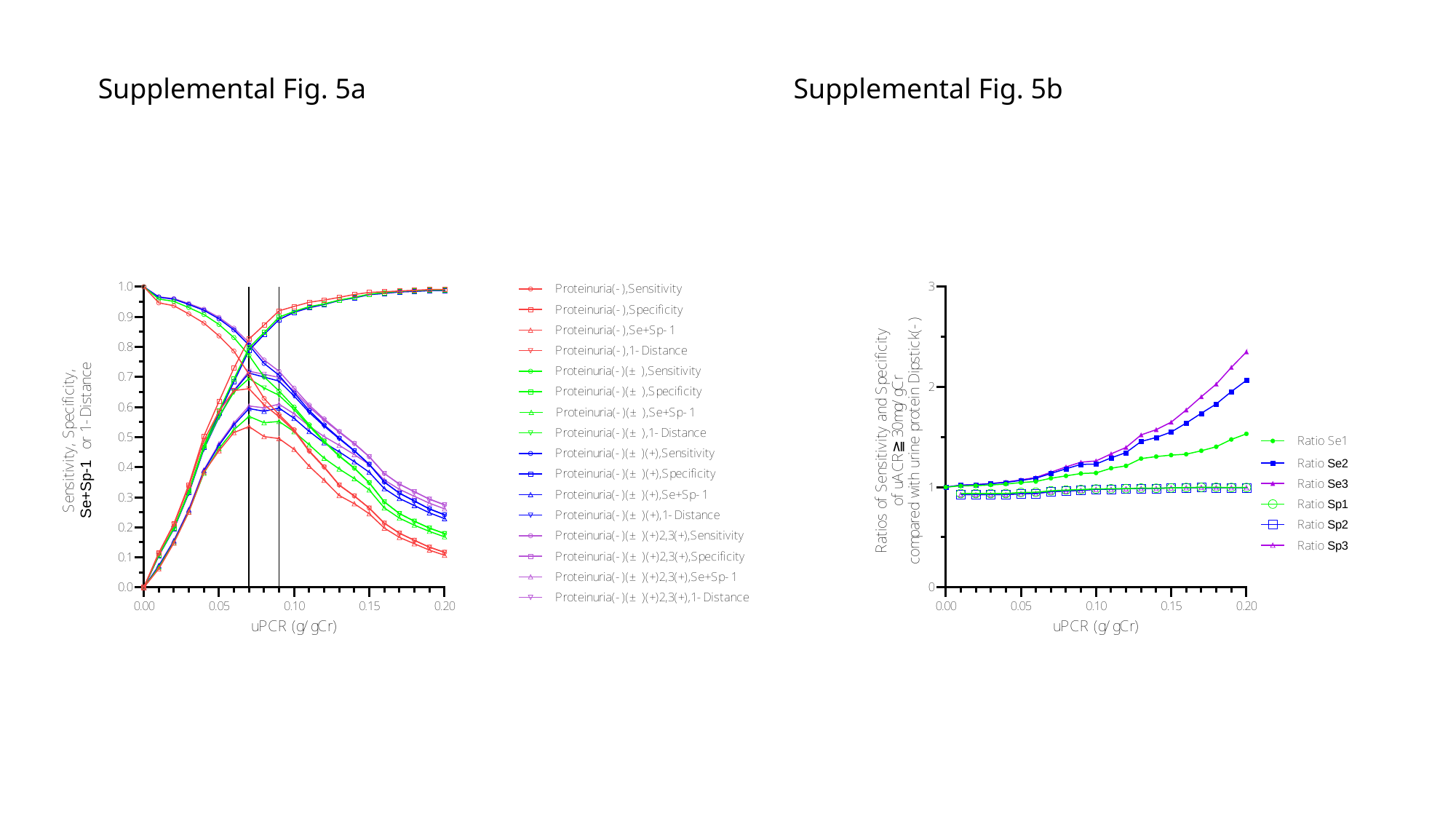

Supplemental Fig. 5a
Supplemental Fig. 5b

## Slide 6
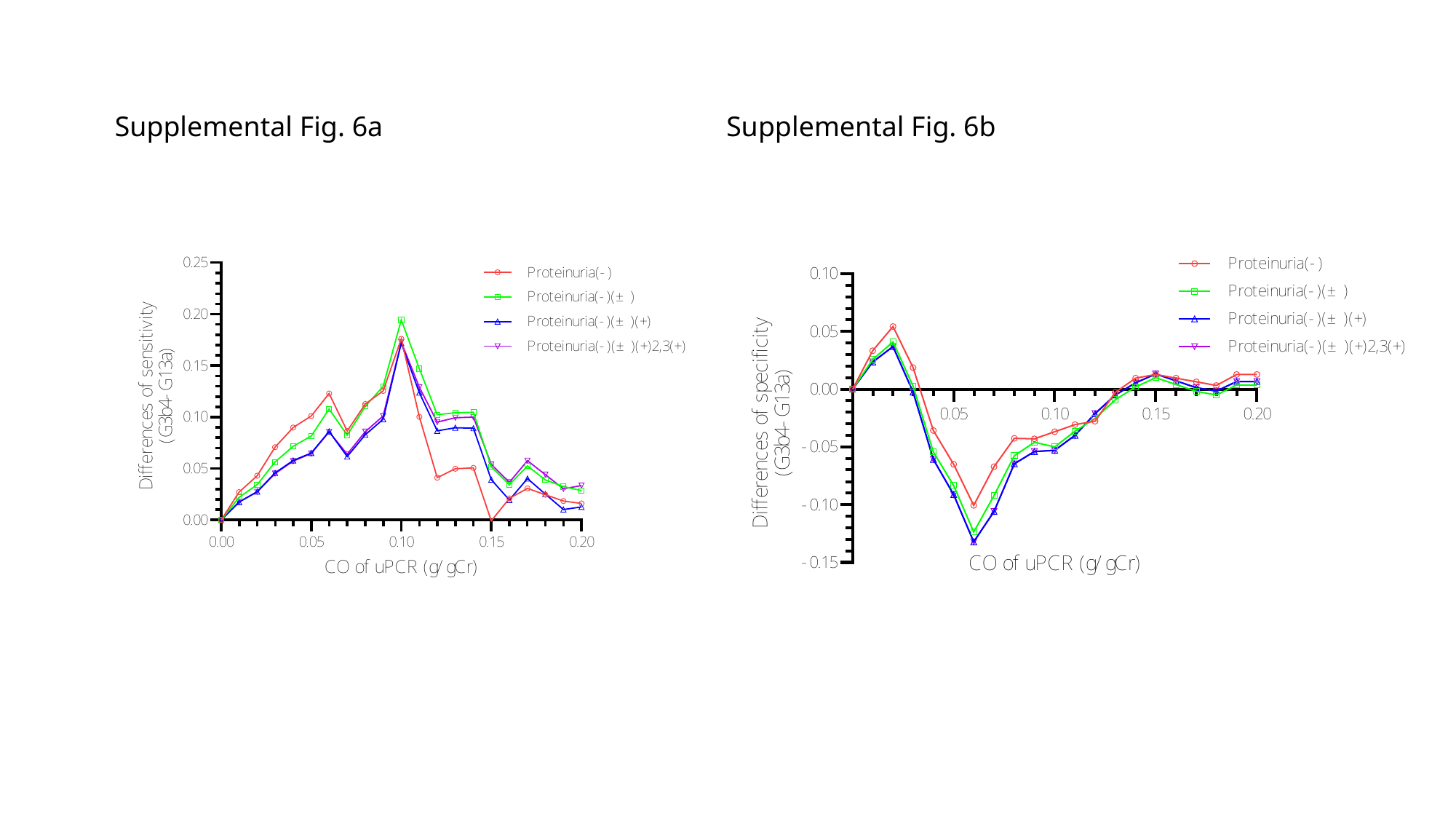

Supplemental Fig. 6a
Supplemental Fig. 6b

## Slide 7
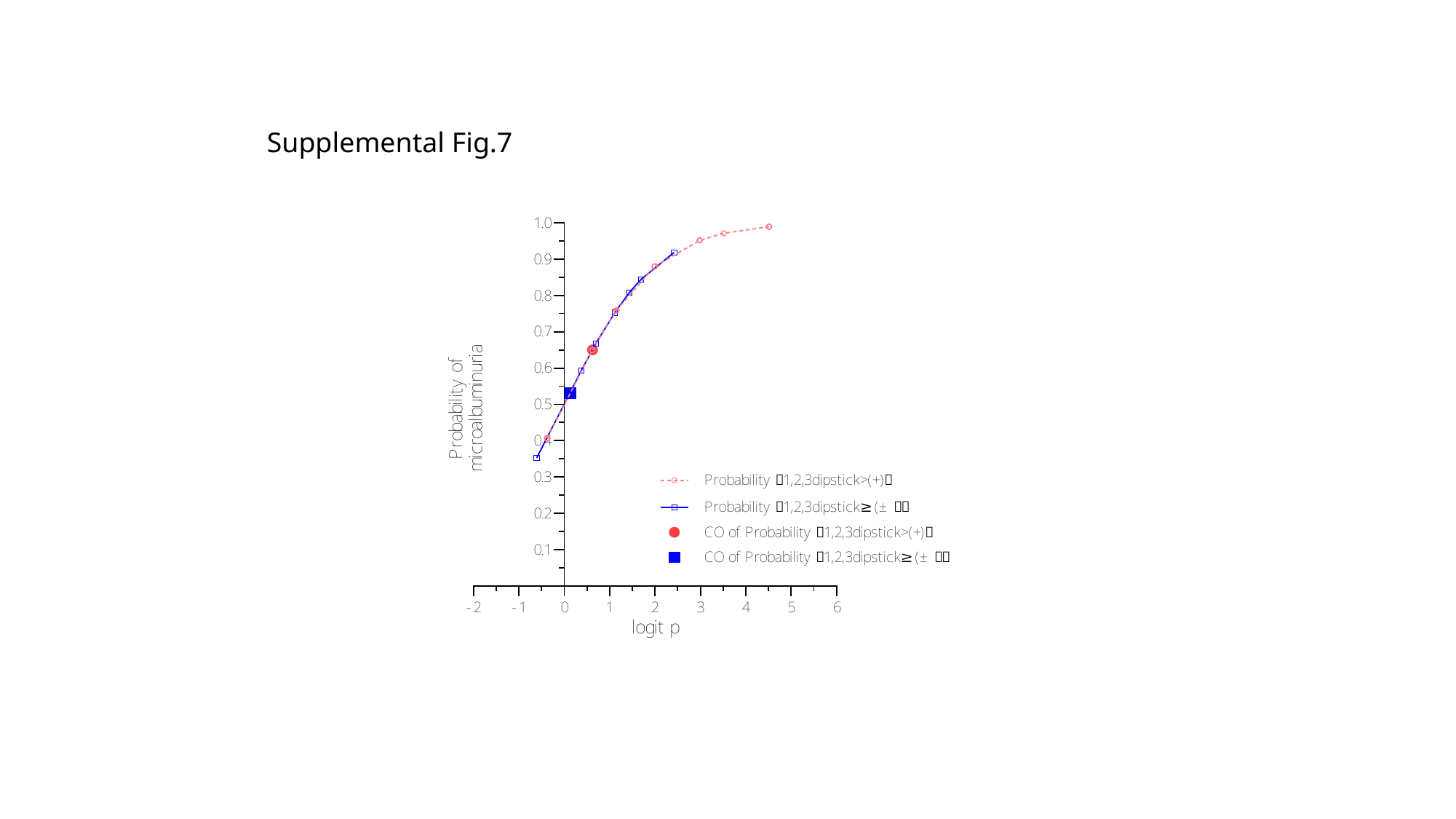

Supplemental Fig.7

## Slide 8
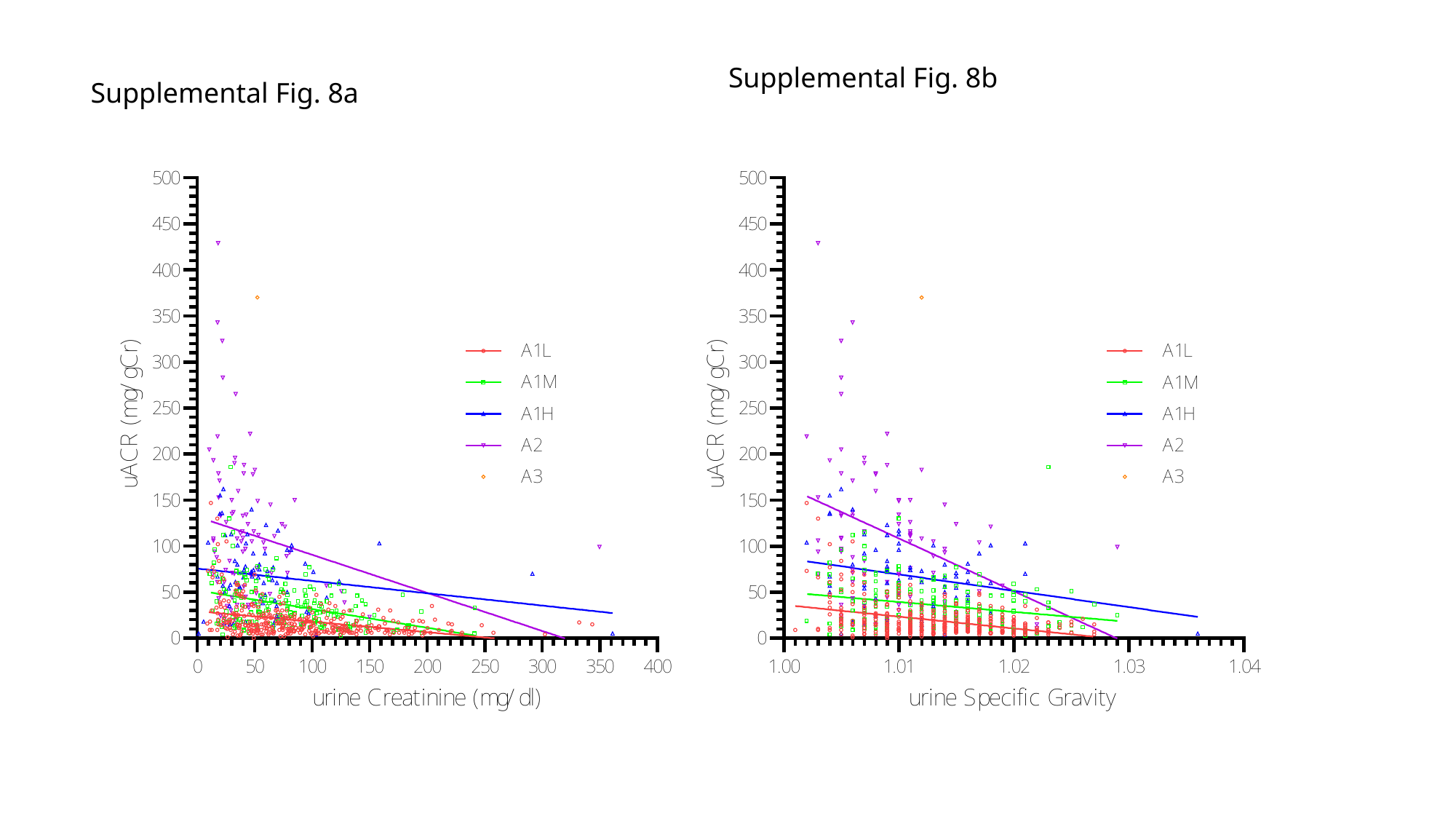

Supplemental Fig. 8b
Supplemental Fig. 8a

## Slide 9
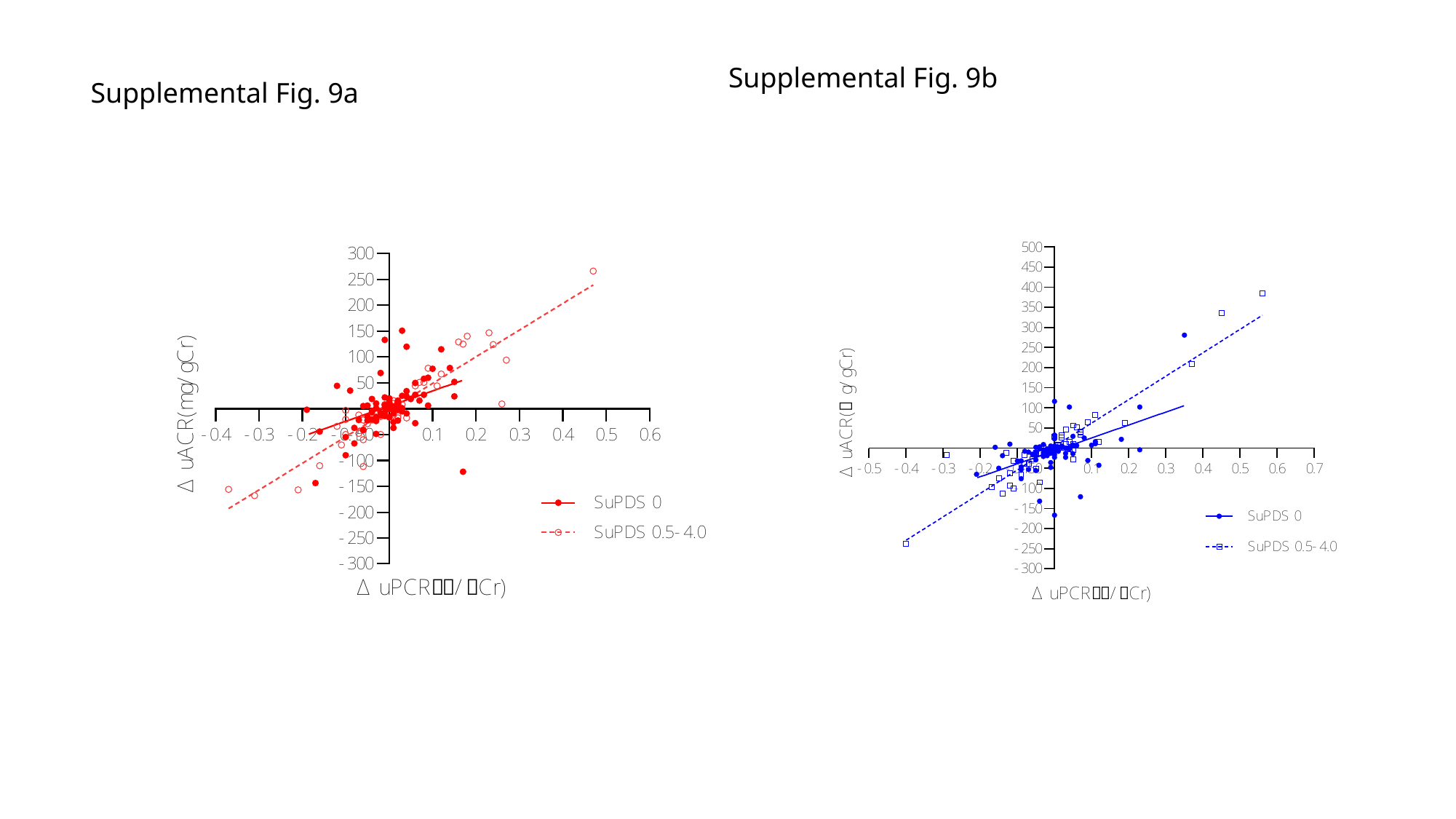

Supplemental Fig. 9b
Supplemental Fig. 9a
